# The release of osteoclast-stimulating factors on supraphysiological loading by osteoprogenitors coincides with expression of genes associated with inflammation and cytoskeletal arrangement

**DOI:** 10.1038/s41598-022-25567-7

**Published:** 2022-12-14

**Authors:** Cornelia Bratengeier, Astrid D. Bakker, Aneta Liszka, Jörg Schilcher, Anna Fahlgren

**Affiliations:** 1 Department of Biomedical and Clinical Sciences, Division of Cell Biology, Linköping University, Linköping, Sweden; 2 Department of Oral Cell Biology, Academic Centre for Dentistry Amsterdam (ACTA), University of Amsterdam and Vrije Universiteit Amsterdam, Amsterdam Movement Sciences, Amsterdam, The Netherlands; 3 Department of Orthopedics and Department of Biomedical and Clinical Sciences, Faculty of Health Sciences and the Wallenberg Centre for Molecular Medicine, Linköping University, Linköping, Sweden

**Keywords:** Deformation dynamics, Extracellular signalling molecules, Differentiation, Targeted bone remodelling

## Abstract

Supraphysiological loading induced by unstable orthopedic implants initiates osteoclast formation, which results in bone degradation. We aimed to investigate which mechanosensitive cells in the peri-implant environment produce osteoclast-stimulating factors and how the production of these factors is stimulated by supraphysiological loading. The release of osteoclast-stimulating factors by different types of isolated bone marrow-derived hematopoietic and mesenchymal stem cells from six osteoarthritic patients was analyzed after one hour of supraphysiological loading (3.0 ± 0.2 Pa, 1 Hz) by adding their conditioned medium to osteoclast precursors. Monocytes produced factors that enhanced osteoclastogenesis by 1.6 ± 0.07-fold and mesenchymal stem cells by 1.4 ± 0.07-fold. Medium from osteoprogenitors and pre-osteoblasts enhanced osteoclastogenesis by 1.3 ± 0.09-fold and 1.4 ± 0.03-fold, respectively, where medium from four patients elicited a response and two did not. Next generation sequencing analysis of osteoprogenitors revealed that genes encoding for inflammation-related pathways and cytoskeletal rearrangements were regulated differently between responders and non-responders. Our data suggest that released osteoclast-stimulating soluble factors by progenitor cells in the bone marrow after supraphysiological loading may be related to cytoskeletal arrangement in an inflammatory environment. This connection could be relevant to better understand the aseptic loosening process of orthopedic implants.

## Introduction

Osteoarthritis is a degenerative joint disease that affects joint cartilage and underlying bone. The most successful medical intervention to provide pain relief and restore function is total joint replacement^1^. Although total joint replacement has good overall long-term survival rates^2^, the implant will undergo aseptic loosening in some patients and require challenging revision surgery^3^. Aseptic loosening is associated with the formation of a “synovium‐like” fibrous tissue membrane around the unstable implant, resulting in small peri-implant gaps and micromotions. These micromotions represent the major predictive factor for early and late implant failure^4–6^. Weight-bearing activities performed on an unstable implant accelerates the fluid within these peri-implant gaps, resulting in a supraphysiological mechanical stimulus. Compared with the physiological loads on healthy bone, this stimulus is several orders of magnitude higher during gait cycles^7^. These supraphysiological mechanical loads can trigger a shift in the production of signaling factors by cells at the peri-implant interface, shifting the balance between bone formation and resorption towards increased bone resorption^8–10^. The exact mechanisms for this shift and why some patients develop aseptic implant loosening^11,12^ and others do not are unknown.

The mechanostat theory suggests that mechanical loading on bone in a specific physiological range helps maintaining skeletal mass, but mechanical loading under or above this range activates bone remodeling that affects the balance between bone-forming osteoclasts and bone-resorbing osteoclasts^13^. These mechanical loads (e.g., tension exerted through the extracellular matrix, compression, or shear stress through the movement of fluids^14^) are recognized by cells through cellular deformation that results in a biochemical response, a process known as mechanotransduction^15,16^. During mechanotransduction, the cellular membrane is strongly affected by cytoskeletal arrangement^17,18^, membrane tension^19^, molecules present in the cell membrane^20,21^, and inflammation^22^. Although osteoarthritis is a non-inflammatory disease, the wear of the joints causes mononuclear cells such as T‑cells and macrophages to invade the synovial membrane and trigger a secondary inflammatory response^23^. This secondary inflammatory response can also affect mechanotransdution of bone cells. For example, osteocytes can produce excessive pro-inflammatory cytokines such as Interleukin (IL-)1β and IL-6 or tumor necrosis factor alpha (TNF‑α), amplifying the inflammatory process and promoting bone degredation^24^. Mechanical loading in a physiological range has potent anti-inflammatory effects as they inhibit the NF-κB signaling cascade^25^, which suppresses osteoclast formation. However, it is unknown how bone cells perceive mechanical loads above the physiological range and convert these supraphysiological mechanical loads into a biochemical response in an inflammatory environment. Furthermore, it is unknown to what extent genes and proteins related to the cytoskeleton and its membrane-associated molecular complexes differ. Therefore, more information is needed to understand why cells in a peri-implant interface do or do not respond to supraphysiological mechanical loads in an inflammatory environment. With these new insights, it might be possible to understand why some patients develop aseptic loosening of orthopedic implants and others do not. This difference may be related to the ability of cells in the peri-implant interface to sense mechanical loads and conduct the mechanotransduction process. It is also unknown which cells at the peri-implant interface produce factors that stimulate bone resorption in response to supraphysiological loading . In addition, the bone marrow contains several bone-related precursor cells from the mesenchymal lineage that differentiate into bone-forming osteoblasts and later into mechanosensitive osteocytes and from the hematopoietic lineage that differentiate into osteoclasts. The effect of mechanical loading on more mature bone cells is well established^26,27^, but the influence of mechanical loading on bone marrow-derived precursor cells from human samples remains unclear.

Although it remains unclear which soluble factors are the main driving factors for the overloading-induced osteoclast formation, we have previously reported how MLO-Y4 osteocyte-like cells and mouse bone marrow-derived hematopoietic progenitor cells respond to supraphysiological loading. MLO-Y4 osteocyte-like cells responded to supraphysiological loading by increasing extracellular nitric oxide (NO), soluble osteoprotegerin (OPG), and membrane-bound receptor activator of nuclear factor kappa-Β ligand (RANKL) but did not affect soluble prostaglandin E2 (PGE2) or soluble RANKL^28^. Mouse bone marrow-derived hematopoietic progenitor cells responded to supraphysiological loading by increasing the release of adenosine triphosphate (ATP)^29^. Although extracellular ATP is considered a potent factor for osteoclast formation and activity^30^, its short life-span might indicate that unknown factors are involved in the supraphysiological loading-induced osteoclast formation. We do not know whether the response is similar in humans.

In this study, we aimed to identify the cell types in the bone marrow that respond to supraphysiological loading and the genes and proteins that might affect the release of osteoclast-stimulating factors by bone marrow cells exposed to supraphysiological loading. We hypothesized that cell types that respond to supraphysiological mechanical loading with the induction of osteoclastogenesis and those which do not predominantly differ in genes and proteins related to inflammation and cytoskeleton rearrangement. To test this hypothesis, we used isolated primary bone marrow cells from patients undergoing total joint replacement. We induced differentiation towards a variety of progenitor and mature bone cells because it is unknown which *human* cell type in the peri-implant interface responds to supraphysiological loading with the induction of osteoclast formation. These cells were subjected to one hour of supraphysiological loading (3.0 ± 0.2 Pa, 1 Hz) through the application of a pulsating fluid flow, and their capacity to release soluble factors that induce osteoclast formation was evaluated.

## Results

### Bone marrow-derived cells from six osteoarthritic patients responded differently to supraphysiological loading

**Figure 1:**
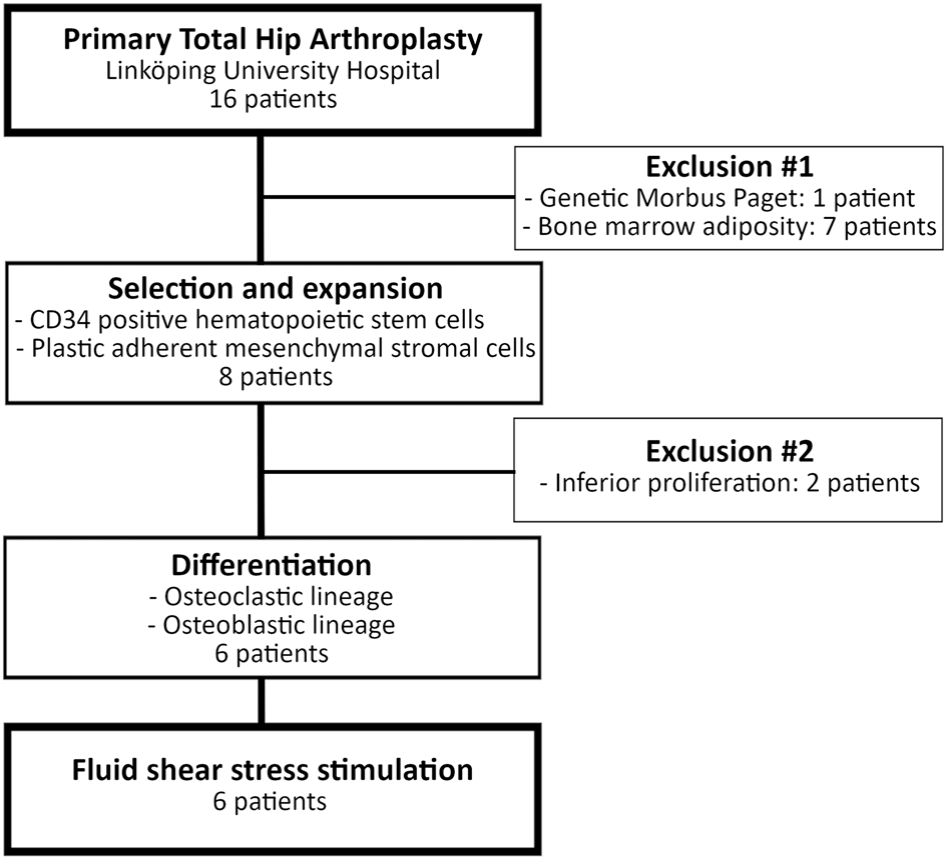
Flowchart showing the selection of samples. From the 16 patients undergoing primary total hip arthroplasty, ten patients had to be excluded from this study as they exhibited genetic Morbus Paget’s disease, predominantly adipose bone marrow, or insufficient proliferation of their isolated stem cells.

Although we received bone samples from 16 osteoarthritic patients, we had to exclude ten patients from the study – seven patients showed a drastically reduced number of isolated bone marrow cells from the enriched cell fraction after density gradient centrifugation due to a predominantly adipose bone marrow content, one patient suffered from genetic Morbus Paget’s disease, and two patients showed inferior proliferation capacities of isolated stem cells during expansion. Samples from six patients fulfilled the two inclusion criteria (Fig. 1, Table 1) — i.e., enough cells in the enriched cell fraction after density gradient centrifugation and good proliferation capacities during cell expansion. Cells from the isolated fractions were expanded and differentiated toward the osteoblastic and osteoclastic lineage as described in section “Expansion of human hematopoietic stem cells (HSC) and induction of osteoclastic differentiation” and in section “Expansion of human mesenchymal stromal cells (MSC) and induction of osteogenic differentiation” in the supplementary information. Stem cells and differentiated progenitor cells that were subjected to supraphysiological loading showed cell type- and patient-specific differences in their capacity to release osteoclast-stimulating soluble factors (Table 1, Supplementary Fig. S1).

**Table 1:** Overview of patients included in this study and their evaluated capacity to respond ( +) or not respond (−) to supraphysiological loading with the release of osteoclast-stimulating soluble factors.

Patient ID	Age	Gender	Mesenchymal stromal cells	Osteoprogenitor	Pre-osteoblasts	Monocytes	Pre-osteoclasts
001H	44	Female	+	+	+	+	**−**
002H	53	Male	+	+	+	+	**−**
004H	54	Female	+	+	+	+	**−**
005H	61	Male	+	+	+	+	**−**
013H	51	Female	+	**−**	**−**	+	**−**
014H	66	Male	+	**−**	**−**	+	**−**

﻿Taken together, we isolated mesenchymal and hematopoietic stem cells from human bone marrow of patients undergoing primary total hip arthroplasty, induced their differentiation into lineage-specific progenitors, and evaluated their capacity to release osteoclast-stimulating soluble factors after supraphysiological loading.

### Gene expression analysis for verification of different differentiation stages in the mesenchymal lineage

**Figure 2:**
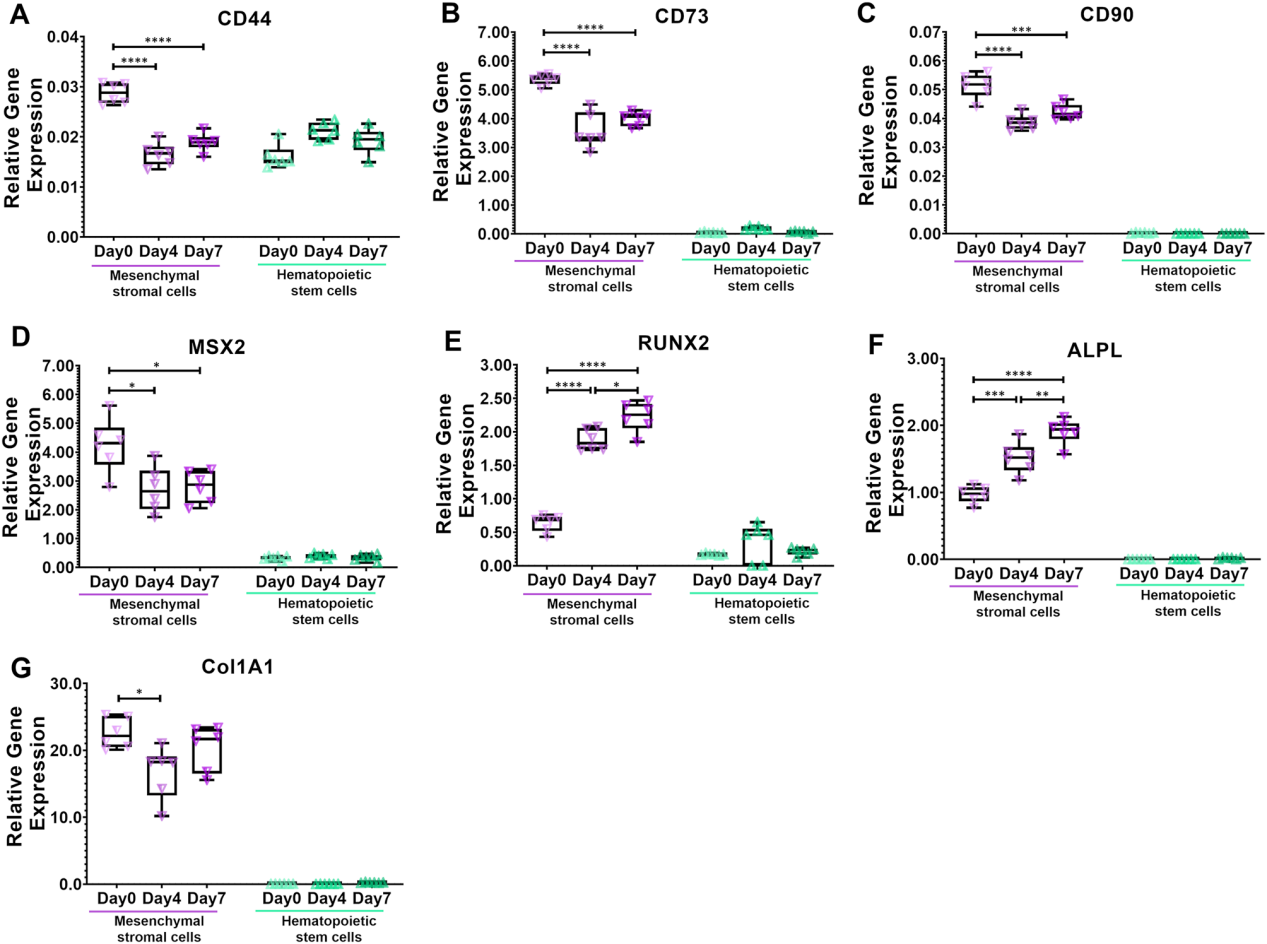
The induction of osteogenic differentiation of mesenchymal stromal cells was verified by gene expression analysis. (**A–D**) Mesenchymal stromal cells (Day 0) were identified by an increased expression of (**A**) Hematopoietic Cell E- And L-Selectin Ligand (CD44), (**B**) 5'-Nucleotidase Ecto (CD73), (**C**) Thy-1 Membrane Glycoprotein (CD90), and (**D**) Homeobox Protein Hox-8 (MSX2). (**E–G**) Induction of osteogenic differentiation into osteoprogenitors (Day 4) and early pre-osteoblasts (Day7) were verified by continuously increasing expression in genes related to osteoblast differentiation: (**E**) Runt-Related Transcription Factor 2 (RUNX2), (**F)** Alkaline Phosphatase Liver/Bone/Kidney Isozyme (ALPL), and Collagen Type I Alpha 1 Chain (Col1A1). *p<0.05, **p<0.01, ***p<0.001, ****p<0.0001, one-way analysis of variance with Bonferroni post hoc test. n=6 individual patients.

The stem cell stage of the mesenchymal stromal cells (day 0) was evaluated. Although gene expression of Hematopoietic Cell E- And L-Selectin Ligand (CD44) was generally low, it was 1.7-fold (p < 0.0001) and 1.5-fold (p < 0.0001) higher in mesenchymal stromal cells (day 0) than in osteo-progenitor cells (day 4) and pre-osteoblasts (day 7), respectively (Fig. 2A). 5'-Nucleotidase Ecto (CD73) was highly expressed in all stages of the mesenchymal lineage, although it was highest in mesenchymal stromal cells compared to osteo-progenitor cells (day 4, 1.5-fold, p < 0.0001) and to pre-osteoblasts (day 7, 1.3-fold, p < 0.0001) (Fig. 2B). Expression of Thy-1 membrane glycoprotein (CD90) was generally low, although it was 1.3-fold (p < 0.0001) and 1.2-fold (p = 0.0008) higher in mesenchymal stromal cells (day 0) than in osteo-progenitor cells (day 4) and pre-osteoblasts (day 7), respectively (Fig. 2C). Homeobox Protein Hox-8 (MSX2) was highly expressed in all stages of the mesenchymal lineage. It was highest in mesenchymal stromal cells compared to osteo-progenitor cells (day 4, 1.6-fold, p = 0.0103) and pre-osteoblasts (day 7, 1.5-fold, p = 0.0165) (Fig. 2D).

The induction of osteogenic differentiation was evaluated by changes in gene expression reported to be involved in the osteoblast differentiation process. Runt-Related Transcription Factor 2 (RUNX2) (Fig. 2E) and Alkaline Phosphatase Liver/Bone/Kidney Isozyme (ALPL) (Fig. 2F) showed a continuous increase in gene expression upon induction of osteogenic differentiation. In osteoprogenitor cells (day 4), RUNX2 was 2.9-fold higher (p < 0.0001) and ALPL was 1.6-fold (p = 0.0004) higher compared to mesenchymal stromal cells (day 0). In pre-osteoblastic cells (day 7), RUNX2 was 3.5-fold (p < 0.0001) higher and ALPL was 2.0-fold (p < 0.0001) higher compared to mesenchymal stromal cells (day 0). Additionally, RUNX2 was 1.3-fold (p = 0.0109) higher and ALPL was 1.2-fold (p = 0.0070) higher compared to osteoprogenitor cells (day 4) (Fig. 2 E, F). Collagen Type I Alpha 1 Chain (Col1A1) was highly expressed in all stages of the mesenchymal lineage, although it was higher in mesenchymal stromal cells compared to osteo-progenitor cells (day 4, 1.3-fold, p = 0.0214) but not compared to pre-osteoblasts (day 7) (Fig. 2G). Altogether, we were able to isolate and expand mesenchymal stem cells from human bone marrow and successfully progressed their differentiation into the osteoblastic lineage.

### Gene expression analysis for verification of different differentiation stages in the hematopoietic lineage

**Figure 3:**
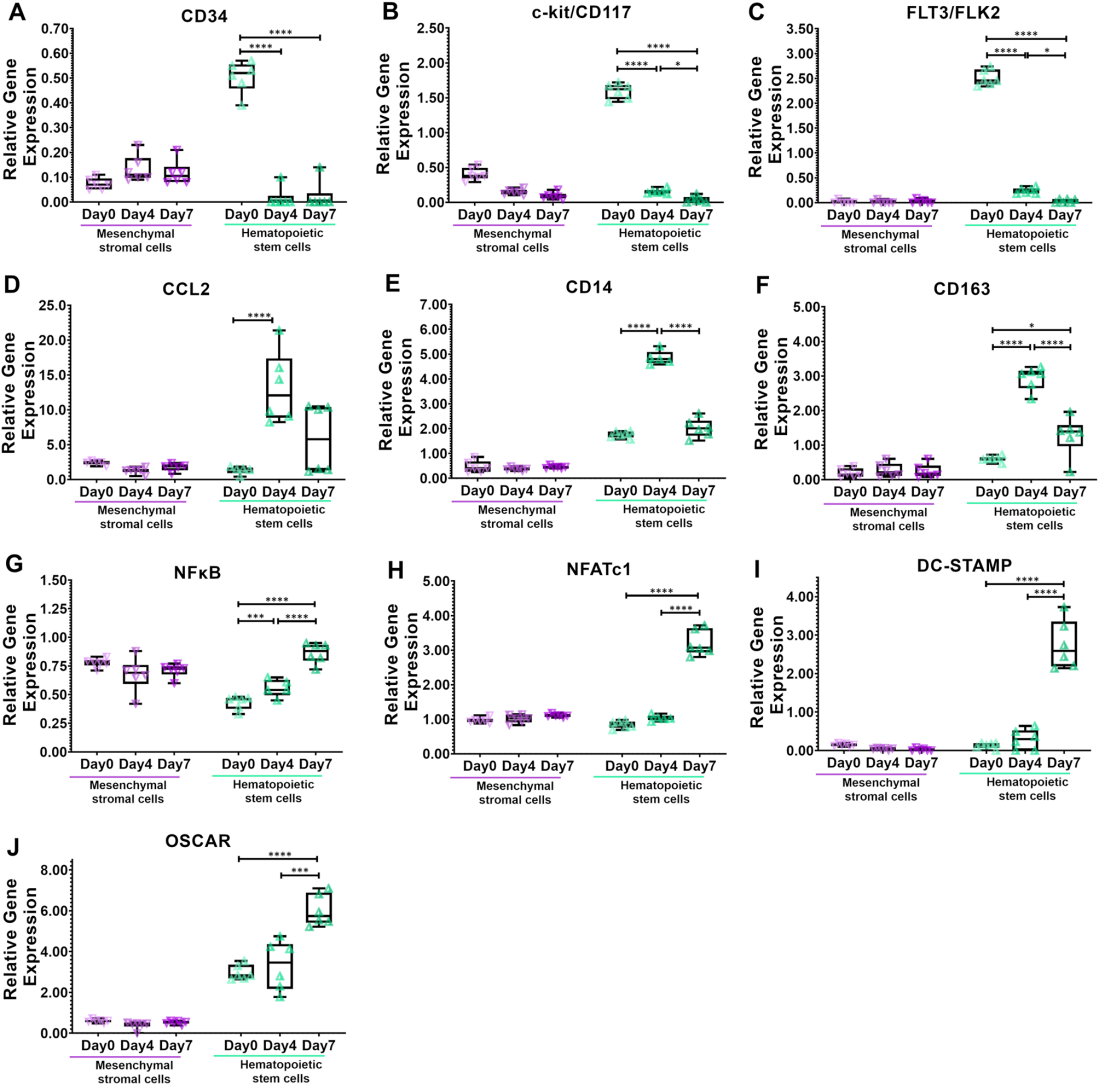
The induction of osteoclastic differentiation of hematopoietic stem cells was verified by gene expression analysis. (**A–C**) Hematopoietic stem cells (Day 0) were identified by an increased expression of (**A**) Hematopoietic Progenitor Cell Antigen (CD34), (**B**) KIT Proto-Oncogene, Receptor Tyrosine Kinase (ckit/CD117), and (**C**) Fms Related Tyrosine Kinase 3 (FLT3 (**D–F**) Induction of osteoclastogenic differentiation into monocytes (Day 4) was verified by an increased expression of (**D**) C–C Motif Chemokine Ligand 2 (CCl2), (**E**) Monocyte Differentiation Antigen (CD14), and (**F**) Scavenger Receptor Cysteine-Rich Type 1 Protein M130 (CD163). (**G–J**) Induction of osteoclastogenic differentiation into pre-osteoclasts (Day 7) was verified by an increased expression of (**G)** Nuclear Factor Kappa B Subunit 2 (NFκB), (**H)** Nuclear Factor Of Activated T Cells 1 (NFATc1), (**I)** Dendritic Cells (DC)-Specific Transmembrane Protein (DC-STAMP), and (**J**) Osteoclast Associated, Immunoglobulin-Like Receptor (OSCAR). *p<0.05, **p<0.01, ***p<0.001, ****p<0.0001, one-way analysis of variance with Bonferroni post hoc test. n = 6 individual patients.

The stem cell stage of hematopoietic stem cells (day 0) was evaluated. Although gene expression of Hematopoietic Progenitor Cell Antigen (CD34) was generally low, it was expressed 30.3-fold (p < 0.0001) higher compared to monocytes (day 4) and 22.2-fold (p < 0.0001) higher compared to pre-osteoclasts (day 7) (Fig. 3A). Expression of KIT Proto-Oncogene, Receptor Tyrosine Kinase (c-kit/CD117) was highest in hematopoietic stem cells (day 0) compared to monocytes (day 4, 10.4-fold, p < 0.0001) and pre-osteoclasts (day 7, 41.1-fold, p < 0.0001) (Fig. 3B). Fms Related Tyrosine Kinase 3 (FLT3/FLK2) was expressed highest in hematopoietic stem cells (day 0) compared to monocytes (day 4, 10.6-fold, p < 0.0001) and pre-osteoclasts (day 7, 71.5-fold, p < 0.0001) (Fig. 3C).

Induction of differentiation to monocytes was evaluated by changes in genes known to be expressed in monocytes. Expression of C–C Motif Chemokine Ligand 2 (CCL2) was highest in monocytes (day 4) compared to hematopoietic stem cells (day 0, 9.8-fold, p = 0.0012) and pre-osteoclasts (day 7, 2.3-fold, p = 0.0291) (Fig. 3D). Monocyte Differentiation Antigen (CD14) was expressed highest in monocytes (day 4) compared to hematopoietic stem cells (day 0, 2.8-fold, p < 0.0001) and pre-osteoclasts (day 7, 2.4-fold, p < 0.0001) (Fig. 3E). A similar expression of Scavenger Receptor Cysteine-Rich Type 1 Protein M130 (CD163) was highest in monocytes (day 4) compared to hematopoietic stem cells (day 0, 4.9-fold, p < 0.0001) and pre-osteoclasts (day 7, 2.3-fold, p < 0.0001) (Fig. 3F). Expression of Nuclear Factor Kappa B Subunit 2 (NFκB) was increased in monocytes compared to hematopoietic stem cells (day 0, 1.3-fold, p = 0.0474) and expression was lower compared to pre-osteoclasts (day 7, 0.6-fold, p < 0.0001) (Fig. 3G).

The differentiation into pre-osteoclasts was evaluated by genes known to be expressed in osteoclasts. Expression of early gene changes on osteoclast differentiation, Nuclear Factor Kappa B Subunit 2 (NFκB) (Fig. 3G), and Nuclear Factor of Activated T Cells 1 (NFATc1) (Fig. 3H) was highest in pre-osteoclasts (day 7) compared to hematopoietic stem cells (day 0, 2.0-fold, p < 0.0001 and 3.8-fold, p < 0.0001, respectively) and monocytes (day 4, 1.5-fold, p < 0.0001 and 3.1-fold, p < 0.0001, respectively). Additionally, expression of Dendritic Cells (DC)-Specific Transmembrane Protein (DC-STAMP) was highest in pre-osteoclasts (day 7) compared to hematopoietic stem cells (day 0, 21.1-fold, p < 0.0001) and monocytes (day 4, 9.6-fold, p < 0.0001) (Fig. 3I). Expression of Osteoclast Associated, Immunoglobulin-Like Receptor (OSCAR) was also highest in pre-osteoclasts (day 7) compared to hematopoietic stem cells (day 0, 2.0-fold, p < 0.0001) and monocytes (day 4, 1.8-fold, p = 0.0002) (Fig. 3J). Altogether, we isolated and expanded hematopoietic stem cells from human bone marrow and successfully progressed their differentiation into the osteoclastic lineage.

### Some human bone marrow-derived cells respond to physiological loading and stress shielding by releasing of osteoclast-modulating soluble factors

After confirming the differentiation stages as described above, isolated and differentiated bone marrow-derived cells from the six patients (Table 1) were exposed for one hour to physiological mechanical loading (0.7 ± 0.3 Pa, 1 Hz) or simulated stress shielding (pathological unloading, 0.0 ± 0.0 Pa, 0 Hz) to evaluate their capacity to release soluble factors that modulate osteoclast formation.

The soluble factors released after physiological loading by mesenchymal stem cells (0.7 ± 0.04-fold, p < 0.0001), osteoprogenitor cells (0.7 ± 0.07-fold, p = 0.008), and pre-osteoblasts (0.7 ± 0.04-fold, p = 0.02) decreased osteoclast formation compared to the positive control in a RANKL-induced osteoclast assay (Supplementary Fig. S2). Simulation of stress shielding (absence of active fluid flow in the chamber) did not trigger the release of osteoclast modulating factors by mesenchymal stem cells, osteoprogenitors, or pre-osteoblasts (Supplementary Fig. S2).

Soluble factors from monocytes subjected to physiological loading decreased osteoclast numbers (0.4 ± 0.06-fold, p = 0.0001), while pre-osteoclasts subjected to physiological loading did not release factors that altered osteoclast numbers (Supplementary Fig. S3). Stress shielding did not trigger the release of osteoclast modulating factors by pre-osteoclasts, but factors from monocytes increased osteoclast formation by 1.6 ± 0.08-fold (p < 0.0001; Supplementary Fig. S3).

All investigated cell types, except pre-osteoclast, are capable of modulating osteoclast formation via released soluble factors in response to physiological mechanical loading.

### Increased stimulation of osteoclast formation by human bone marrow-derived cells subjected to supraphysiological loading depends on cell type and patient

After investigating the capacity of bone marrow-derived cells to respond to physiological mechanical loading, we exposed them to supraphysiological loading to evaluate their capacity to release soluble factors that increase the stimulation of osteoclast formation when exposed to extreme mechanical loading as can be found around a loosening orthopedic implant.

**Figure 4:**
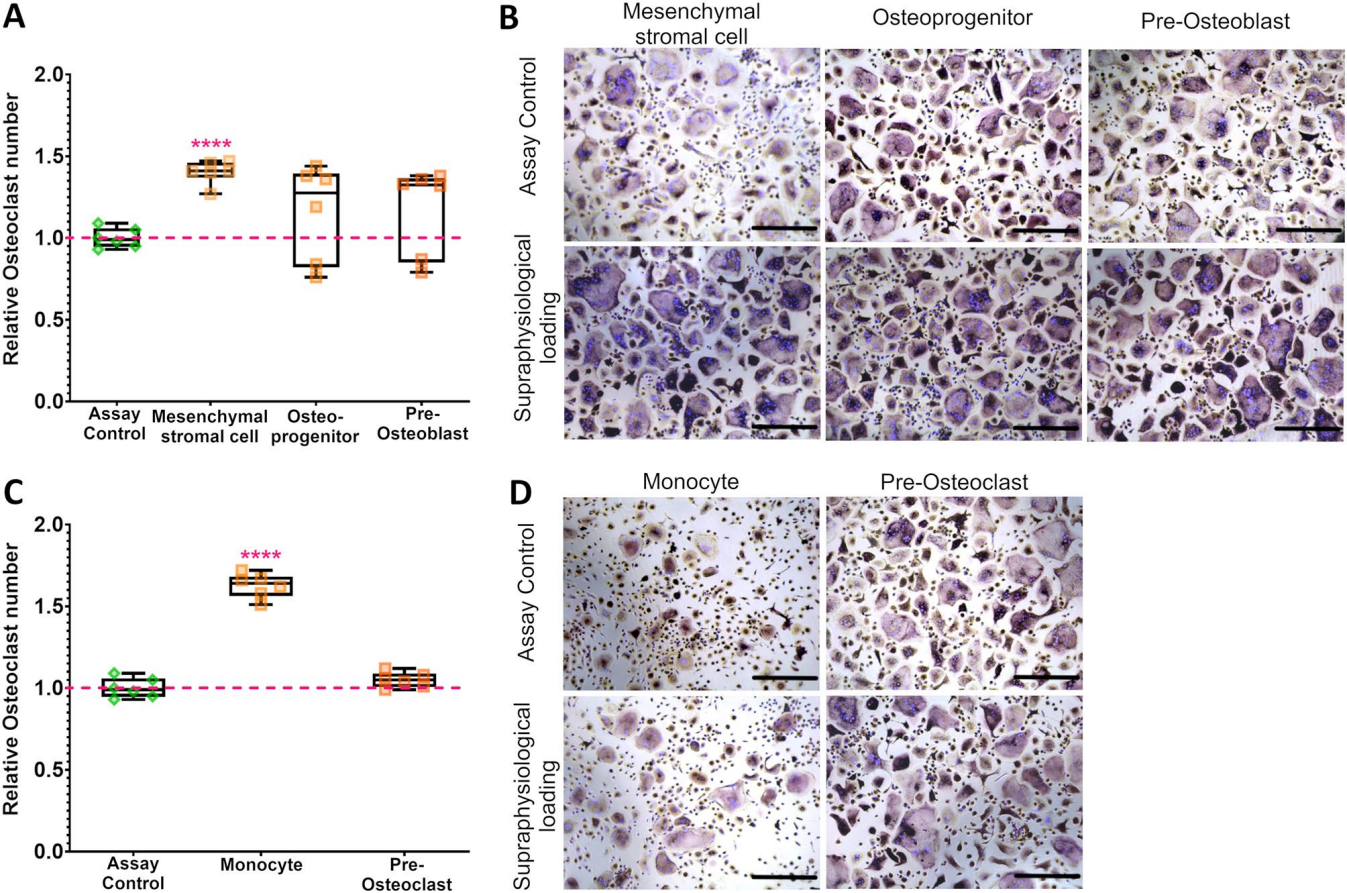
Induction of osteoclast formation via soluble factors upon supraphysiological loading. (**A**) Supraphysiological loading induces osteoclast formation by mesenchymal stem cells, while osteo-progenitor and pre-osteoblasts show patient-specific differences in the response. In both cases, two patients (ID #013H and ID #014H) did not induce osteoclast formation (i.e., below the red line). (**C**) Supraphysiological loading induces osteoclast formation by monocytes, but pre-osteoclasts do not induce osteoclast formation. (**B,D**) Representative images displaying the induction of osteoclast-formation in a RANKL-induced osteoclast assay after 10 days of incubation with the conditioned medium from a responder. ****p < 0.0001, one-way ANOVA with Bonferroni post-hoc test. n = 6 individual patients. Scale bars: 200 µm.

In the mesenchymal lineage, supraphysiological loading of mesenchymal stem cells for one hour induced the release of soluble factors, leading to a 1.4 ± 0.07-fold (p < 0.0001) increase in the number of osteoclasts compared to the positive control in a RANKL-induced osteoclast assay. Supraphysiological loading applied to osteoprogenitor cells and pre-osteoblasts led to an individual response, where four patients induced osteoclast formation by 1.3 ± 0.09-fold (p < 0.0001) and 1.3 ± 0.02-fold (p < 0.0001), respectively. The osteoprogenitor cells and pre-osteoblasts of the two remaining patients (in both cases ID #013H and ID #014H) did not produce factors that lead to increased osteoclast formation (Fig. 4A,B).

In the hematopoietic lineage, supraphysiological loading for 1 h on monocytes induced the release of osteoclast-stimulating soluble factors, leading to a 1.6 ± 0.07-fold (p < 0.0001) increase in the number of osteoclasts compared to the assay positive control. Pre-osteoclasts did not respond to supraphysiological loading with the release of osteoclast-stimulating soluble factors (Fig. 4C,D).

Therefore, mesenchymal stem cells and monocytes from all donors released soluble factors that greatly contribute to an increased osteoclast formation upon supraphysiological loading. Interestingly, a patient-specific response could be observed in osteoprogenitors and pre-osteoblasts. Cells from four patients (responders) produced factors that stimulate osteoclast formation upon supraphysiological loading, but osteoprogenitors and pre-osteoblasts from two patients (non-responders) failed to produce factors that stimulate osteoclast formation upon supraphysiological loading.

### Next-generation RNA sequencing and validation by RT-qPCR

We further investigated the puzzling observation that osteoprogenitors and pre-osteoblasts from patients could be separated into responders and non-responders in terms of their capacity to release soluble factors that induce osteoclast formation upon supraphysiological loading. Could it be that they differ in their ability to sense mechanical loading or their ability for mechanotransduction? Therefore, we analyzed osteoprogenitors from both responders and non-responders to investigate their genetic makeup via next-generation RNA sequencing.

**Figure 5:**
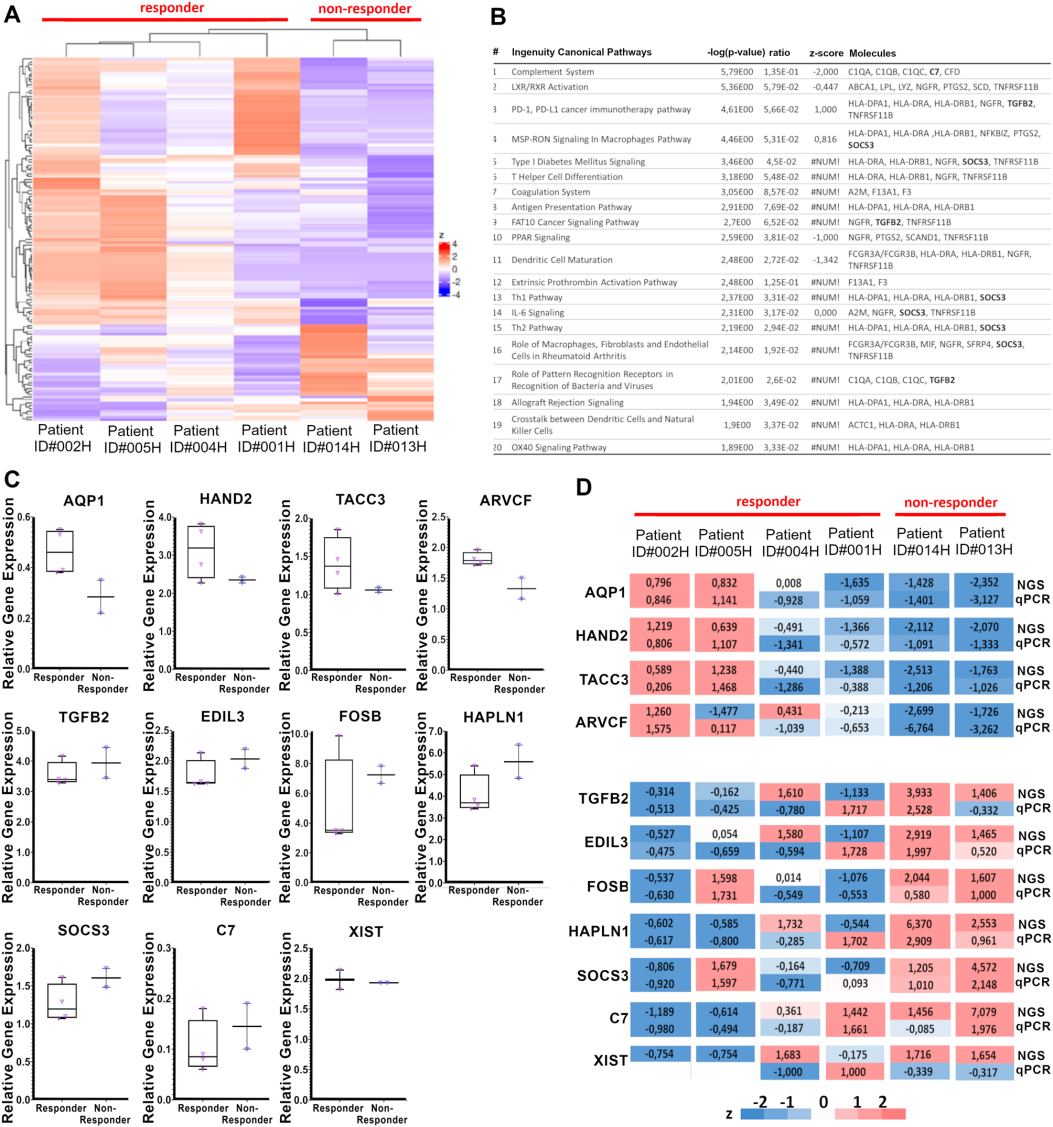
Osteoprogenitors from non-responders show differentially expressed pathways and gene patterns, validated by RT-qPCR analysis. (**A**) The raw hybridization data were normalized and subjected to trended dispersion calculation. After standardization (z) to mean = 0 and SD = 1, genes with differential expression (FDR p-value < 0.1) were subjected to hierarchical clustering heatmap analysis, revealing upregulated (red) and downregulated (blue) genes in the osteoprogenitors of individual patients, clearly separating the two non-responding patients (ID#013H and #014H). (**B**) The top 20 differentially regulated pathways in non-responders were identified by Ingenuity Pathways Analysis. Despite the lack of active regulation in some pathways (z-score of #NUM), all the top-regulated pathways (except for #9, FAT10 Cancer Signaling Pathway) were directly linked to T-helper cell differentiation, activation, and function. (**C**) RT-qPCR was performed on 11 selected genes. (**D**) To compare the RNA sequencing data (NGS) and RT-qPCR gene expression analysis (qPCR) between the two groups, we used standardization(z) to mean = 0 and SD = 1 to visualize the upregulated (red) and downregulated (blue) genes.

In total, 12,471 differentially expressed genes were identified using next generation RNA sequencing analysis. Applying analysis cut-offs (FDR p-value < 0.1, ± 0.5-fold change) using QIAGEN IPA, we reduced the number of statistically significantly differentially expressed genes to 127. A hierarchical clustering heatmap of the 127 genes revealed that the expression pattern was similar between the two non-responders. The resulting grouping separated the two non-responders from the four responders (Fig. 5A). Among the 127 differentially expressed genes, 229 unique pathways were identified (Supplementary Table S1). The top 20 of these pathways (Fig. 5B) showed strong connections to inflammation, innate/adaptive immune responses, or autoimmune responses in the Ingenuity Target Explorer (https://targetexplorer.ingenuity.com/index.htm, QIAGEN). Overall, 19 of the 20 pathways were strongly linked to T-helper cell differentiation, activation, and function.

Of the 127 differentially expressed genes, 11 were selected for RT-qPCR analysis based on showing the strongest downregulation or upregulation in the two non-responders. Analysis with RT-qPCR of selected genes showed similar expression profiles to those seen in the next-generation RNA sequencing experiments. AQP1 (0.62 ± 0.15-fold), HAND2 (0.75 ± 0.02-fold), TACC3 (0.76 ± 0.02-fold), and ARVCF (0.75 ± 0.09-fold) showed tendencies towards lower expression in the osteoprogenitors of non-responders compared to responders. Similarly, TGFB2 (1.11 ± 0.14-fold), EDIL3 (1.15 ± 0.09-fold), FOSB (1.44 ± 0.12-fold), HAPLN1 (1.38 ± 0.19-fold), SOCS3 (1.27 ± 0.09-fold), and C7 (1.41 ± 0.45-fold) showed tendencies towards higher expression in the osteoprogenitors of non-responders compared to responders. Of the 11 genes tested, only XIST did not show the regulation pattern in osteoprogenitors expected from the RNA sequencing data (Fig. 5C). These similarities were also demonstrated when the z-scores were applied to the mean RT-qPCR data to describe the regulation patterns of gene expression between non-responders and responders (Fig. 5D).

## Discussion

The cellular membrane acts as a mechanosensor where alterations in gene expression greatly influence the membrane’s physical properties^31,32^ (e.g., membrane tension), altering its mechanism of mechanotransduction^19^. How these alterations contribute to the capacity of bone cells to release osteoclast-stimulating soluble factors after supraphysiological loading remains elusive. Using bone marrow-derived stem cells and their lineage-specific progenitor cells, we encountered a deviation in the release of osteoclast-stimulating soluble factors in cells of either specific patients or more progressed differentiation stages. Upon further investigation of this non-responder phenomenon, we encountered a deviation in the genetic makeup from cells of non-responding patients that prevented the release of osteoclast-stimulating soluble factor after supraphysiological loading.

The physical properties of the cellular membrane and resulting viscoelasticity characteristics of a cell can determine its capacity to respond to mechanical loading^33^. However, it is poorly understood how variations in genetic levels, potentially linked to continuous inflammation that modulate the viscoelastic properties of cells, affect the release of osteoclast-stimulating soluble factors in bone cells of osteoarthritic patients. To resolve this question, we looked further into the observation that osteoprogenitors and pre-osteoblasts of some patients responded to supraphysiological loading with induction of osteoclast formation, while the corresponding cells of other patients did not. Next generation RNA sequencing analysis revealed a genetic makeup within osteoprogenitors that might affect cytoskeletal rearrangements, potentially diminishing their capability to sense mechanical stimuli or to transduce mechanical stimuli to a biochemical signal.

Among the top 20 regulated pathways, we found that the majority were associated with T-cell differentiation, activation, or action despite a lack of active regulation in some pathways. T-cells contribute to the progression of loading-induced osteoarthritis^34^, and specific subsets of T-helper cells (Th1 cells, Th9 cells, Th17 cells, and follicular helper T cells) and cytotoxic T cells are correlated with the severity of osteoarthritis^35^. The severity of osteoarthritis is characterized by bone-resorption, often in response to the infiltration of inflammatory cells such as T cells into the synovial membranes^36^. In bone, certain subsets of T-cells—i.e., regulatory T (Treg) cells and T helper 17 (Th17) cells—directly influence bone homeostasis. Although Treg cells and Th17 cells both require the TGF-β regulated signaling pathway for differentiation, they counteract each other in terms of bone homeostasis^37,38^: Treg cells promote bone formation through secretion of osteoblast-stimulation Wnt10b and prevent osteoclast formation through secretion of granulocyte–macrophage colony-stimulating factor (GM-CSF), interferon-γ (IFN-γ), IL-5, and IL-10^39,40^. However, Th17 stimulates osteoclast formation through RANKL/RANK signaling^41^ and block osteoblasts through secretion of TNF-α, IL-1, and IL-6^42^. Local inflammatory mediators can directly affect bone remodeling^43,44^, but the effects of these inflammatory mediators on the behavior of the bone cell as a mechanotransducer remains poorly understood. Although inflammatory cytokines can affect cellular behaviors^45^, we have seen this effect only in response to supraphysiological loading, not physiological loading. It is possible that the physiological stimulus might curb inflammation by suppressing the actions of inflammatory mediators^46^. However, examining other cell types offers a potential explanation for the lack of response. For example, in human epithelial cells, the inflammatory process in relation to C-reactive proteins affects mechanotransduction through increased membrane stiffness^47^ as a result of F-actin interaction^48,49^ and therefore cytoskeletal regulation^50^.

The cytoskeleton is a dynamic structure and is critical for bone cells to maintain their shape and function. For example, mechanotransduction^51^ can regulate the reorganization of the cytoskeleton^52^. Inflammatory markers have also been reported to affect cytoskeletal rearrangement in many cell types. In macrophages, inflammatory activation by either lipopolysaccharide (LPS) and interferon gamma (IFNγ) results in a biphasic response where dynamic reorganization of actin and myosin initiates a contractile phase followed by a later spreading phase^53^. In addition, the activation of the complement system has shown to directly interact with intermediate filaments in monocytes, leading to their degredation^54^ and a disruption of the cellular membrane^55^. This is noteworthy as the complement system was the top-differently regulated pathway when analyzing osteoprogenitors of responders and non-responders. In endothelial cells, inflammatory mediators increase the influx of calcium, which results in an increased cellular permeability through disassembling of adherent junctions and cytoskeletal rearrangement^56^. This increased cellular permeability is also mediated through a disruption of the balance between cytoskeletal contractile forces and actin-myosin engagement adhesive forces^57^. Similarly, the importance of calcium influx in bone cells in response to mechanical loading and the resulting effect on cytoskeletal regulations have been documented^58^. This response and our findings indicate that a genetic makeup in osteoarthritic patients with increased inflammatory markers could prime bone cells and diminish their capacity to either sense supraphysiological loading or convert the supraphysiological stimulus to a biological signal–i.e., the release of osteoclast-stimulating soluble factors. Interestingly, the connection to a shift in membrane stiffness through cytoskeletal rearrangement and membrane tension is similarly evident when focusing on the top downregulated and upregulated genes.

There are certain limitations to this study. Although we included bone-marrow-derived cells from a clinically relevant group of patients, our inclusion criteria resulted in a stringently selected patient pool with the best cellular proliferation qualities in vitro. Thus, the obtained results are potentially susceptible to selection bias. Furthermore, we identified two patients who did not induce osteoclast formation upon supraphysiological loading when differentiating their mesenchymal stem cells towards osteoprogenitors and pre-osteoblasts, further constraining our findings. Thus, it is critical to validate these findings with a higher number of non-responding cell types or patients. Furthermore, we did not evaluate the response to supraphysiological loading for CD34+ hematopoietic stem cells because they are unable to attach to a fibronectin-coated surface.

In conclusion, the genes related to increased inflammation and cytoskeletal rearrangement might prevent the release of osteoclast-stimulating soluble factors after supraphysiological loading. This was coherent with results obtained from the genetic analysis of differently regulated pathways and genes of osteoprogenitors from non-responding patients. To confirm that these findings can be directly translated to aseptic loosening of total joint replacements will require further follow-up studies with a larger set of non-responding patients and an emphasis on clinical outcomes. Nevertheless, we have provided a tantalizing clue that environmental changes (e.g., increased inflammation and cytoskeletal arrangements) might affect the mechanosensory capabilities of the cellular membrane in bone cells in response to supraphysiological loading and its associated release of osteoclast-stimulating soluble factors.

## Material and methods

### Isolation of bone marrow cells from femoral neck bone marrow

Whole femoral heads were collected from 16 patients who underwent primary total joint replacement at Linköping University Hospital between September 2017 and May 2018. Six patient samples fulfilled the inclusion criteria (Table 1, Fig. 1). None of the patients had been diagnosed previously with bone metabolic conditions or had been treated with drugs affecting bone metabolism. As previously described, human mesenchymal stromal cells^59^ and hematopoietic stem cells^60^ were isolated from the enriched cell fraction after density gradient centrifugation. These cells were isolated by scraping off pieces of the trabecular bone of the femoral neck at the level of the osteotomy with a mixing spatula (HWL 010-05, Karl Hammacher GmbH, Germany) after storage for a maximum of one-hour post-surgery at 4 °C in Iscove’s Modified Dulbecco’s Medium (IMDM, Gibco#21980032) supplemented with 2% Fetal Bovine Serum (FBS, Biowest#S1810) and 2-mM Ethylenediaminetetraacetic acid (EDTA, Sigma-Aldrich Sweden AB). Cells were rinsed off the trabecular bone pieces with sterile PBS. The cell suspension was filtered through a 70-µm cell strainer and centrifuged at 200×*g* for 10 min at 4 °C. Enriched cell fraction after density gradient centrifugation was obtained by density gradient centrifugation with Histopaque (1.077 g/ml; Sigma-Aldrich Sweden AB #H8889) and stored at − 150 °C in freezing medium that contained 90% FBS and 10% dimethyl sulfoxide (DMSO; Sigma-Aldrich Sweden AB).

### In vitro model to mimic supraphysiological loading

From the frozen cells collected from the enriched cell fraction after density gradient centrifugation, CD34+ hematopoietic stem cells and plastic adherent mesenchymal stem cells were expanded and differentiated towards the osteoclastic lineage or osteoblastic lineage (Supplementary information). Human stem cells and lineage-committed progenitors were seeded on a 2.2 × 2.2 bovine fibronectin-coated glass slide (Sigma-Aldrich Sweden AB # F4759) at a density of 7200 cells/cm^2^ and subjected to pulsating fluid flow (PFF) using a parallel-plate flow chamber, as described previously^29,61^. For this, fluid flow medium (8 ml) that consisted of MEMα (Gibco#22561021) supplemented with 1% Antibiotic–antimycotic (PSF, Gibco#15240062) was used. Conditioned medium (CM) was collected after 1 h, sterile-filtered through a 0.22-µm cellulose acetate membrane, and stored at − 20 °C.

### Gene expression analysis

Cells from each lineage-specific differentiation stage (Day 0, Day 4, and Day 7) were harvested in 700 µl of TRIzol reagent (Ambion#15596026) and stored at − 80 °C per the manufacturer’s instructions. The concentration and potential protein and phenol contamination (260/280 ratio) of the isolated mRNA were tested in the NanoDrop™ spectrophotometer (Saveen & Werner) with the ND-1000 software (Fisher Scientific). The manufacturer’s instructions were followed to synthesize cDNA using a high-capacity cDNA reverse transcription kit (Applied Biosystems# 4368813). The gene expression levels of markers of osteogenic and osteoclastic differentiation were used to validate the differentiation stage and the genes selected for verification of the RNA sequencing analysis of osteoprogenitor cells from patients who induced osteoclast formation upon supraphysiological loading (responders). Patients who failed to induce osteoclast formation upon supraphysiological loading (non-responders) were investigated. The housekeeping genes 18SrRNA and B2M (mesenchymal lineage) as well as HPRT1 and YWHAZ (hematopoietic lineage) were quantified as they showed stable expression during differentiation (Table 2). Gene expression analysis was performed using the 7500 Fast Real-Time PCR System (Applied Biosystems). A standard curve of serially diluted cDNA from either human brain or human spleen (Invitrogen #AM7962 and #QS0627) was used to correct for PCR efficiency. The expression of genes of interest was normalized against the square root of the product formed by the housekeeping genes in the corresponding lineages.

**Table 2:** Overview of markers used for the gene expression analysis to verify differentiation within the hematopoietic lineage or mesenchymal lineage and the top (up- and down-regulated) differentially expressed genes detected in the next generation RNA sequencing analysis of osteoprogenitor cells from responders and non-responders to supraphysiological loading.

Gene name	Gene symbol	Assay ID
Hematopoietic Progenitor Cell Antigen CD34	CD34	Hs02576480_m1
KIT Proto-Oncogene, Receptor Tyrosine Kinase	c-kit/CD117	Hs00174029_m1
Fms-Related Tyrosine Kinase 3	FLT3/FLK2	Hs00174690_m1
Monocyte Chemoattractant Protein 1	CCL2	Hs00234140_m1
Scavenger Receptor Cysteine-Rich Type 1 Protein M130	CD163	Hs00174705_m1
Monocyte Differentiation Antigen CD14	CD14	Hs02621496_s1
Nuclear Factor-κB Subunit 2	NFKb2	Hs00174517_m1
Nuclear Factor of Activated T Cells 1	NFATc1	Hs00542675_m1
Osteoclast-Associated, Immunoglobulin-Like Receptor	OSCAR	Hs01100185_m1
Dendritic Cells (DC)-Specific Transmembrane Protein	DC-STAMP	Hs00229255_m1
Hematopoietic Cell E- and L-Selectin Ligand	CD44	Hs01075864_m1
5'-Nucleotidase Ecto	CD73	Hs00159686_m1
Thy-1 Membrane Glycoprotein	CD90	Hs00174816_m1
Homeobox Protein Hox-8	MSX2	Hs00741177_m1
Runt-Related Transcription Factor 2	RUNX2	Hs00231692_m1
Alkaline Phosphatase Liver/Bone/Kidney Isozyme	ALPL	Hs01029144_m1
Collagen Type I Alpha 1 Chain	Col1A1	Hs00164004_m1
Aquaporin 1	AQP1	Hs01028916_m1
Heart and neural crest derivatives expressed 2	HAND2	Hs00232769_m1
Transforming acidic coiled-coil containing protein 3	TACC3	Hs01099874_m1
Armadillo repeat gene deleted in velocardiofacial syndrome	ARVCF	Hs01554141_m1
Transforming growth factor β2	TGFB2	Hs00234244_m1
EGF-like repeats and discoidin domains 3	EDIL3	Hs00964112_m1
FosB proto-oncogene, AP-1 transcription factor subunit	FOSB	Hs00171851_m1
X inactive-specific transcript (non-protein coding)	XIST	Hs01079824_m1
Hyaluronan and proteoglycan link protein 1	HAPLN1	Hs01091999_m1
Suppressor of cytokine signaling 3	SOCS3	Hs02330328_s1
Complement component 7	C7	Hs00940408_m1
18S Ribosomal RNA	18S rRNA	Hs03003631_g1
Beta-2-Microglobulin	B2M	Hs00187842_m1
Hypoxanthine Phosphoribosyltransferase 1	HRPT1	Hs02800695_m1
Tyrosine 3-Monooxygenase/Tryptophan 5-Monooxygenase Activation Protein Zeta	YWHAZ	Hs01122445_g1

### RANKL-induced osteoclastogenesis assay

Based on the well-established protocol for human osteoclast differentiation assay^62^, peripheral blood mononuclear cells (PBMC) from the whole blood of one voluntary donor (GeBlod.se, Linköping, Sweden) were isolated from peripheral blood using density gradient centrifugation with Histopaque 1.077 (Sigma-Aldrich Sweden AB). The cells were then incubated at 37 °C with 5% CO_2_ with 20 ng/ml human Macrophage Colony-Stimulating Factor (hMCSF; R&D Systems# 216-MC) for 4 days. Attached myeloid progenitor cells were harvested with 0.25% Trypsin–EDTA (Gibco# 25200056), and 30,000 cells per well were cultured in a mixture of 50% freshly prepared medium and 50% conditioned medium obtained after fluid shear stress, simulating physiological loading, supraphysiological loading, and stress shielding. Each well contained MEMα with a final concentration of 10% FBS, 1% PSF, 20 ng/ml hMCSF, and 20 ng/ml human soluble receptor activator of NF-κB ligand (hsRANKL, PeproTech Nordic#462-TEC). A positive control was performed with 100% fresh culture medium supplemented with 20 ng/ml hMCSF and 20 ng/ml hsRANKL, while in the negative control hsRANKL was omitted. The experiment was kept at 37 °C with 5% CO_2_ and medium was changed every 3–4 days. On Day 10, cells were fixed with 4% formaldehyde and stained for tartrate-resistant acid phosphatase (TRACP) per the manufacturer’s instructions (Sigma-Aldrich Sweden AB#386A-1KT). The numbers of TRACP-positive multinucleated cells (≥ 3 nuclei/cell) were manually counted using the Axio Vert.A1 fluorescence microscope with the N-Achroplan 10×/0.45 M27 objective lens (Carl Zeiss AB).

### Whole-transcriptome analysis with total RNA sequencing

The concentration and RNA integrity number (RIN; ID#001: 8.7, ID#002: 7.2, ID#004: 9.1, ID#005: 9.4, ID#013: 9.0, and ID#014: 8.5) of the isolated mRNA samples were evaluated using the Agilent 2100 Bioanalyzer (Agilent Technologies Sweden AB) instrument with the Agilent RNA 6000 Nano reagent kit (Agilent Technologies#5067-1511). No contamination of genomic DNA was detected by agarose gel electrophoreses. Samples were stored at − 80 °C before RNA sequencing.

Transcriptome analysis using next generation RNA sequencing was performed by the core facility at Novum, BEA (Karolinska Institute). Briefly, total RNA was subjected to quality control with Agilent Tapestation (Agilent Technologies) per the manufacturer’s instructions. To construct libraries suitable for Illumina sequencing, we used the Illumina TruSeq Stranded mRNA sample preparation (Illumina# 20020595) protocol, which includes mRNA isolation, cDNA synthesis, ligation of adapters, and amplification of indexed libraries. The yield and quality of the amplified libraries were analyzed using Qubit (Fisher Scientific) and the Agilent Tapestation (Agilent Technologies). The indexed cDNA libraries were normalized and combined, and the pools were sequenced in the Illumina NextSeq 550 (Illumina Inc.) for a 75-cycle v2 sequencing run that generated 75-bp single-end reads.

Bioinformatics support for data analysis was provided by the core facility at Novum, BEA. The obtained lists of differentially expressed genes for the combination of non-responding patients and the combination of the remaining four responding patients were generated by a trended dispersion calculation. This list was used in the QIAGEN Ingenuity Pathway Analysis (QIAGEN IPA, https://digitalinsights.qiagen.com) for pathway and gene analysis, applying a False Discovery Rate (FDR) p-value cutoff of 0.1 and a fold-change cutoff of higher/lower than 0.5.

### Identification of differentially expressed genes by total RNA sequencing in individual patients for RT-qPCR verification

Differentially expressed genes after RNA sequencing in the osteoprogenitors of each patient were used to identify target genes for verification of the results of the RT-qPCR. Raw counts for all six patients were used, and the reads per kilobase of the transcript per million reads (RPKM) were applied for transcript quantification using Eq. (1), taking the single-read sequencing method and RNA molecule length into consideration ^63^:

1 $$RPKM=\frac{Number\, of \,reads\, mapped\, to\, gene\, \times \,{10}^{9}}{Total\, number\, of \,mapped \,reads\,\times \,gene \,length\, in\, bp}$$

Z-scores (Eq. 2) were calculated from the quantified RPKM transcript quantifications for each patient and used to evaluate the differential expression of genes from the two patients who did not induce osteoclast formation (non-responders), compared with the four patients who did induce osteoclast formation (responders), upon supraphysiological loading. Responders were set as the reference as follows:

2 $$z{\rm -}score=\frac{Observed \,value\, -Mean \,of \,the\, reference\, sample }{Standard \,deviation\, of\, the\, reference \,sample}$$

The top regulated genes were identified according to z-score (6 genes for downregulation and 10 genes for upregulation) in the two non-responders. These genes were compared to the list of 127 differentially expressed genes (FRD < 0.1, fold-change cutoff higher/lower than 0.5) obtained from the QIAGEN IPA gene analysis. To be considered for RT-qPCR validation, genes were required to show stronger regulation (down- or up-) according to the z-score in the non-responders than in the responders. The identified genes were further investigated for their functions using the Gene Ontology (GO) knowledgebase (http://geneontology.org/) and a literature review using the Web of Science core collection database (https://apps.webofknowledge.com). In the review, we focused on three key functions: (1) involvement in cytoskeleton arrangement and actin remodeling; and (2) effects in bone that specifically influenced osteoblast/osteoclast activity or differentiation. Genes with a connection to at least one of these two key functions were selected for RT-qPCR validation, which resulted in a list of four downregulated genes and seven upregulated genes (Table 3). After normalization to the housekeeping genes, z-scores were calculated using Eq. (2). This calculation allowed us to directly compare the differential expression patterns of the RT-qPCR and RNA sequencing data.

**Table 3:** Z-score of genes identified by next generation RNA sequencing with the strongest downregulated and upregulated genes in non-responding patients and their affiliation with either cytoskeleton arrangement and actin remodeling or influencing osteoblast/osteoclast activity or differentiation.

Ensembl	Entrez	Symbol	Length	z-score						Cyto-skeletal/actin remodeling	Effect on bone cells
				Responder				Non-responder			
				Patient ID #002H	Patient ID #005H	Patient ID #004H	Patient ID #001H	Patient ID #014H	Patient ID #013H		
ENSG00000160949	4796	TONSL	7107	− 0.819	1.711	− 0.475	− 0.417	− 3.489	− 2.978	** − **	** − **
ENSG00000240583	358	**AQP1**	3436	− 1.635	0.796	0.008	0.832	− 2.352	− 1.428	+	+
ENSG00000164107	9464	**HAND2**	3601	− 1.366	1.219	− 0.491	0.639	− 2.070	− 2.112	+	+
ENSG00000013810	10460	**TACC3**	8202	− 1.388	0.589	− 0.440	1.238	− 1.763	− 2.513	+	** − **
ENSG00000099889	421	**ARVCF**	5636	− 0.213	1.260	0.431	− 1.477	− 1.726	− 2.699	+	** − **
ENSG00000141449	80000	GREB1L	10,572	− 1.653	0.274	0.342	1.037	− 1.584	− 1.402	** − **	** − **
ENSG00000092969	7042	**TGFB2**	6050	− 1.133	− 0.314	1.610	− 0.162	1.406	3.933	+	+
ENSG00000164176	10085	**EDIL3**	5825	− 1.107	− 0.527	1.580	0.054	1.465	2.919	** − **	+
ENSG00000125740	2354	**FOSB**	5553	− 1.076	− 0.537	0.014	1.598	1.607	2.044	** − **	+
ENSG00000229807	7503	**XIST**	25,266	− 0.175	− 0.754	1.683	− 0.754	1.654	1.716	** − **	+
ENSG00000144802	64332	NFKBIZ	5902	− 1.258	− 0.537	0.389	1.406	1.663	2.166	** − **	+
ENSG00000145681	1404	**HAPLN1**	6198	− 0.544	− 0.602	1.732	− 0.585	2.553	6.370	+	** − **
ENSG00000164619	168667	BMPER	7990	0.338	− 0.951	1.488	− 0.875	3.013	2.450	** − **	+
ENSG00000094963	2327	FMO2	6201	− 0.016	− 0.300	1.547	− 1.231	4.500	1.147	** − **	** − **
ENSG00000184557	9021	**SOCS3**	2734	− 0.709	− 0.806	− 0.164	1.679	4.572	1.205	+	+
ENSG00000112936	730	**C7**	6682	1.442	− 1.189	0.361	− 0.614	7.079	1.456	+	** − **

Genes marked in bold were chosen for RT-qPCR verification.

### Statistical analysis

One-way analysis of variance (ANOVA) with Bonferroni correction was conducted in the GraphPad Prism 8 for Windows, ver. 8.0.1(224) software (GraphPad Inc.) to determine the statistical significance of the gene expression analysis, to verify the differentiation stages of the mechanically loaded cells, and to determine the modulation of osteoclast formation in relation to the assay control. Formal quantitative statistical analysis was not performed on the genes selected for the RT-qPCR validation of RNA-sequencing data due to the small sample size of non-responders (n = 2). Instead, the central tendency was used to describe the central position within the two datasets: patients who responded to supraphysiological loading (responders) and patients who did not respond to supraphysiological loading (non-responders). In addition, the z-score was used to evaluate the pattern of regulation of genes in individual patients. Data shown are mean ± SD. A p-value < 0.05 was considered to be statistically significant.

### Institutional review board statement

The study was conducted in accordance with the Declaration of Helsinki and approved by the Ethics Committee of Linköping University (#2014/102-31).

### Informed consent statement

Informed consent was obtained from all subjects involved in the study. Written informed consent has been obtained from the patient(s) to publish this paper.

## Supplementary Information

Supplementary Information.

## Data Availability

The data that support the findings of this study are available on request from the corresponding author. The data are not publicly available due to privacy or ethical restrictions.
