# Supplementary material for: The release of osteoclast-stimulating factors on supraphysiological loading by osteoprogenitors coincides with expression of genes associated with inflammation and cytoskeletal arrangement
Source: Sci Rep. 2022 Dec 14;12:21578. doi: 10.1038/s41598-022-25567-7 (PMC9751069; doi:10.1038/s41598-022-25567-7)
Supplement: Supplementary file 1 — Supplementary Information. [file 41598_2022_25567_MOESM1_ESM.docx]

**Overview on the study design**

Bone marrow cells from patients undergoing total joint replacement were retrieved from femoral heads. Enriched cell fraction was collected after density gradient centrifugation. Hematopoietic stem cells were positively selected by magnetic beads sorting for the surface marker CD34, while mesenchymal stem cells were selected by plastic adherence for 48 hours. Both, hematopoietic stem cells and mesenchymal stem cells were expanded and further differentiated towards monocytes and pre-osteoclasts or osteoprogenitors and pre-osteoblasts, respectively. The stage of differentiation was verified by gene expression analysis before cells were subjected to supraphysiological loading for one hour and induction of osteoclast formation was evaluated in a RANKL-induced osteoclastogenesis assay. There, patients were identified that induced osteoclast formation (responders) and patients that failed to induce osteoclast formation (non-responders). Next-generation RNA-sequencing was performed on osteoprogenitors to investigate if genetic variations can be detected that could explain the observed patient-specific differences in osteoclast formation. Statistically significant differentially expressed genes (n=127) were identified and eleven genes were selected for verification by gene expression analysis. The genetic variation in pathway and gene-level could be linked to alterations that affected membrane stiffness (Figure S1).

**
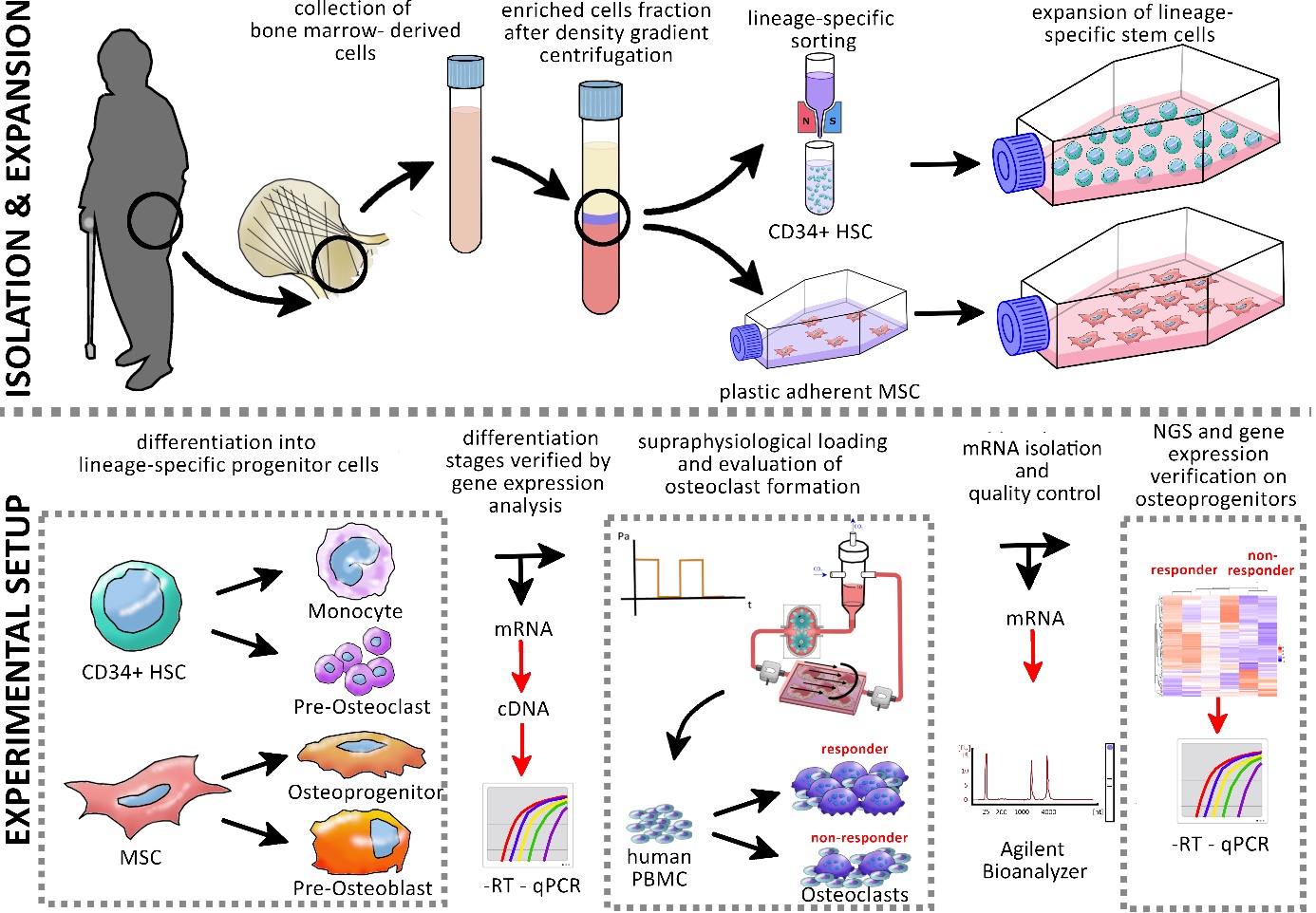
**

**Figure S1. Graphical display of the study design.** Bone marrow-derived CD34+ hematopoietic stem cells (HSC) and plastic adherent mesenchymal stem cells (MSC) were expanded and differentiated into the osteoclastic or osteoblastic lineage. Supraphysiological loading was applied for one hour and the release of osteoclast-modulating soluble factors was evaluated, where responding and non-responding patients were identified. Subsequent next-generation RNA sequencing revealed a connection with the osteoclast-inducing response.

**Expansion of human hematopoietic stem cells (HSC) and induction of osteoclastic differentiation**

From frozen cells collected from the enriched cell fraction after density gradient centrifugation, CD34+ cells were positively selected using human CD34 UltraPure MicroBead Kit (Miltenyi Biotec) according to the manufacturer’s instructions. 1x105 CD34+ cells/ml were cultured in MEMα supplemented with 10% FBS, 1% PSF, 75ng/ml recombinant human SCF/c-kit ligand protein (R&D Systems), 30ng/ml recombinant human Thrombopoietin protein (TPO, R&D Systems), and 75ng/ml recombinant human Flt-3 ligand protein (R&D Systems). The non-adherent cell fraction was transferred after centrifuged at 200×g, 10 minutes at 4°C to fresh medium every third day. After 12 days, expanded CD34+ cells were stored at -150°C in freezing medium containing 90% FBS (Biowest) and 10% DMSO (Merck), until further use. 1.5x105 cells/ml CD34+ cells were used to induce differentiation into monocytes (20ng/ml recombinant human M-CSF Protein (hMCSF, R&D Systems) for 4 days) or pre-osteoclasts (20ng/ml hMCSF for 4 days (R&D Systems) followed by 3 days of 20ng/ml hMCSF (R&D Systems) and 20ng/ml recombinant Human sRANK Ligand (hsRANKL, E.coli derived, Peprotech)). Culture medium was Minimum Essential Medium α (MEMα, Gibco) supplemented with 10% FBS (Biowest) and 1% Antibiotic-Antimycotic (PSF, Gibco). Monocytes (day 4) or pre-osteoclasts (day 7) harvested with 0.25% Trypsin/EDTA (Gibco) and prepared for mechanical stimulation.

**Expansion of human mesenchymal stromal cells (MSC) and induction of osteogenic differentiation**

From frozen cells collected from the enriched cell fraction after density gradient centrifugation, 80000 cells/cm2 were seeded in Minimum Essential Medium α (MEMα, Gibco) supplemented with 10% FBS (Biowest), 1% Antibiotic-Antimycotic (PSF, Gibco), and 1% Glutamax-I Supplement (Gibco). After 48h, non-adherent cells were removed and plastic adherent MSCs were expanded for 14 days, changing the medium twice per week. Expanded MSCs were harvested with 0.05% Trypsin-EDTA (Gibco) for 4 minutes at 37°C and stored at -150°C in freezing medium, containing 90% FBS (Biowest) and 10% DMSO (Merck), until further use. Expanded MSCs were seeded at a density of 2000 cells/cm2 for further expansion and subsequent osteogenic differentiation. Osteogenic differentiation was induced after reaching 60-80% confluency by supplementing culture medium (Minimum Essential Medium α (MEMα, Gibco), 10% FBS (Biowest), 1% Antibiotic-Antimycotic (PSF, Gibco)) with 10mM β-Glycerophosphate (Sigma), 50µg/ml L-ascorbic acid (Arcos organics) and 100nM Dexamethasone (Sigma, water-soluble), changing medium three times a week. MSCs (day 0), osteoprogenitors (day 4) and pre-osteoblasts (day 7) were harvested with 0.25% Trypsin-EDTA (Gibco) and prepared for mechanical stimulation.

**Cells in the mesenchymal lineage secrete osteoclast-reducing soluble factors after 60 minutes physiological loading, while stress shielding did not induce the release of osteoclast-modulating soluble factors.**

In the mesenchymal lineage, mesenchymal stromal cells, osteoprogenitor cells, and pre-osteoblasts were exposed to physiological loading and stress shielding for one hour and released soluble factors were investigated in a RANKL-induced osteoclastogenesis assay.

Sixty minutes of physiological loading on mesenchymal stromal cells (day0), osteoprogenitors (day4) and pre-osteoblasts (day7) induced the release of osteoclast-reducing soluble factors that suppressed the number of osteoclasts by 0.7-fold compared to assay positive control (#OC 117.3±5.6) (Figure S2 A, B). Stress shielding on mesenchymal stromal cells (day0), osteoprogenitors (day4) and pre-osteoblasts (day7) did not change osteoclastogenesis compared to the assay positive control (Figure S2 C, B).
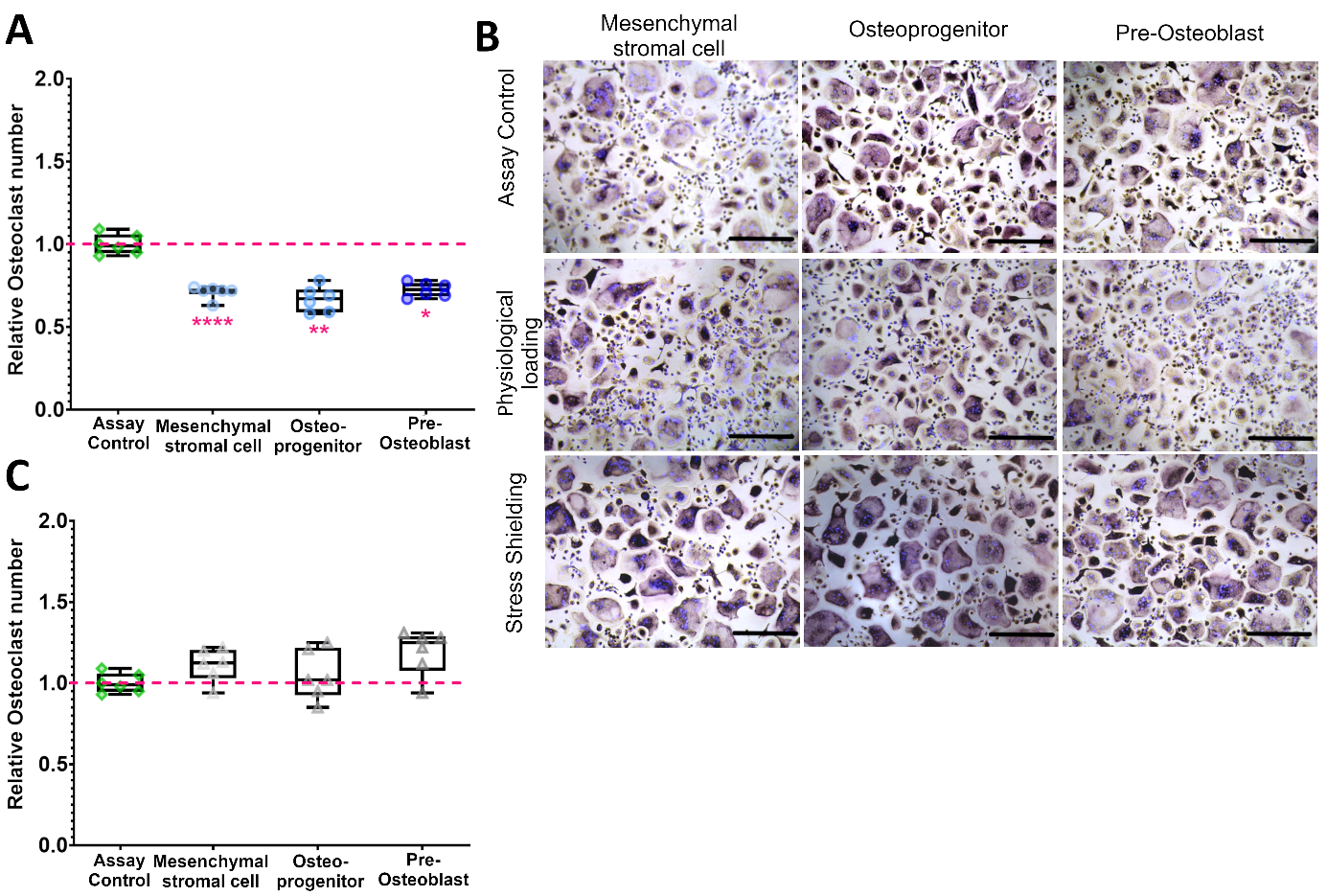


**Figure S2. Cell in the mesenchymal lineage release soluble osteoclast-reducing factors after sixty minutes of physiological loading, while stress shielding had no effect.** [A] Physiological loading induced the released osteoclast-reducing soluble factors in mesenchymal stromal cells, osteoprogenitor, and pre-osteoblasts. [C] Stress shielding on cells in the mesenchymal lineage did not result in the release of osteoclast-modulating soluble factors. [B] Representative images displaying modulation of osteoclast-formation in a RANKL-induced osteoclast assay after 10 days incubation with conditioned medium of a responding patient. *p<0.05, **p<0.01, ****p<0.001, one-way analysis of variance with Bonferroni post hoc test. n=6 individual patients. Scale bars: 200µm

**Monocytes release osteoclast-reducing soluble factors after 60 minutes physiological loading and stress shielding, while pre-osteoclasts did not.**

In the hematopoietic lineage, monocytes and pre-osteoclasts were exposed to physiological loading and stress shielding for one hour and released soluble factors were investigated in a RANKL-induced osteoclastogenesis assay.

Physiological loading for sixty minutes on monocytes (day4) induced the release of osteoclast-reducing soluble factors leading to a 0.4-fold suppression of osteoclast numbers compared to the assay positive control (#OC 83.0±3.9) (Figure S3 A, B). Monocytes (day4) exposed for stress shielding released osteoclast-inducing factors leading to a 1.5-fold increase in the number of osteoclasts compared to assay-positive control. Pre-osteoclasts (day7) did not respond with the release of osteoclast-modulating soluble factors by physiological loading nor stress shielding. (Figure S3 C, B).


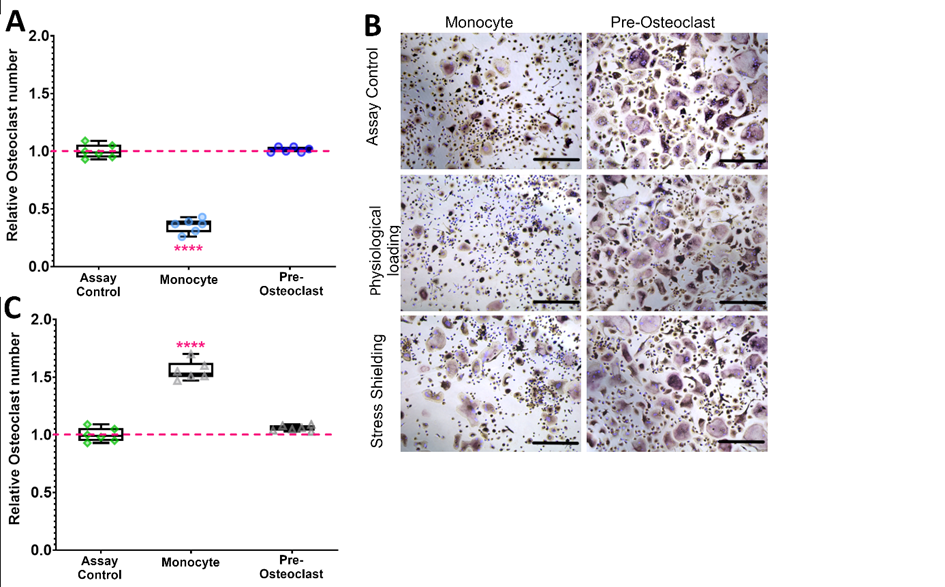


**Figure S3. Monocytes release soluble osteoclast-modulating factors after sixty minutes of physiological loading and stress shielding.** [A] Physiological loading induced the release of osteoclast-reducing soluble factors in monocytes, while pre-osteoclasts did not alter osteoclastogenesis . [C] Stress shielding induced the release of osteoclast-reducing soluble factors in monocytes, while pre-osteoclasts did not alter osteoclastogenesis. [B] Representative images displaying modulation of osteoclast-formation in a RANKL-induced osteoclast assay after 10 days incubation with conditioned medium. ****p<0.001, one-way analysis of variance with Bonferroni post hoc test. n=6 individual patients. Scale bars: 200µm

Table S1: Pathways identified which are associated with the 127 differentially expressed genes identified in non-responders compared to responds in QIAGEN IPA, when analysis cut-offs FDR p-value <0.1 and ±0.5-fold change was applied.

| © 2000-2020 QIAGEN. All rights reserved. | | |  |  |  |  |
| --- | --- | --- | --- | --- | --- | --- |
| **Ingenuity Canonical Pathways** | **-log(p-value)** | **Ratio** | | | **z-score** | **Molecules** |
| Complement System | 5,79E00 | 1,35E-01 | | | -2,000 | C1QA,C1QB,C1QC,C7,CFD |
| LXR/RXR Activation | 5,36E00 | 5,79E-02 | | | -0,447 | ABCA1,LPL,LYZ,NGFR,PTGS2,SCD,TNFRSF11B |
| PD-1, PD-L1 cancer immunotherapy pathway | 4,61E00 | 5,66E-02 | | | 1,000 | HLA-DPA1,HLA-DRA,HLA-DRB1,NGFR,TGFB2,TNFRSF11B |
| MSP-RON Signaling In Macrophages Pathway | 4,46E00 | 5,31E-02 | | | 0,816 | HLA-DPA1,HLA-DRA,HLA-DRB1,NFKBIZ,PTGS2,SOCS3 |
| Type I Diabetes Mellitus Signaling | 3,46E00 | 4,5E-02 | | | #NUM! | HLA-DRA,HLA-DRB1,NGFR,SOCS3,TNFRSF11B |
| T Helper Cell Differentiation | 3,18E00 | 5,48E-02 | | | #NUM! | HLA-DRA,HLA-DRB1,NGFR,TNFRSF11B |
| Coagulation System | 3,05E00 | 8,57E-02 | | | #NUM! | A2M,F13A1,F3 |
| Antigen Presentation Pathway | 2,91E00 | 7,69E-02 | | | #NUM! | HLA-DPA1,HLA-DRA,HLA-DRB1 |
| FAT10 Cancer Signaling Pathway | 2,7E00 | 6,52E-02 | | | #NUM! | NGFR,TGFB2,TNFRSF11B |
| PPAR Signaling | 2,59E00 | 3,81E-02 | | | -1,000 | NGFR,PTGS2,SCAND1,TNFRSF11B |
| Dendritic Cell Maturation | 2,48E00 | 2,72E-02 | | | -1,342 | FCGR3A/FCGR3B,HLA-DRA,HLA-DRB1,NGFR,TNFRSF11B |
| Extrinsic Prothrombin Activation Pathway | 2,48E00 | 1,25E-01 | | | #NUM! | F13A1,F3 |
| Th1 Pathway | 2,37E00 | 3,31E-02 | | | #NUM! | HLA-DPA1,HLA-DRA,HLA-DRB1,SOCS3 |
| IL-6 Signaling | 2,31E00 | 3,17E-02 | | | 0,000 | A2M,NGFR,SOCS3,TNFRSF11B |
| Th2 Pathway | 2,19E00 | 2,94E-02 | | | #NUM! | HLA-DPA1,HLA-DRA,HLA-DRB1,SOCS3 |
| Role of Macrophages, Fibroblasts and Endothelial Cells in Rheumatoid Arthritis | 2,14E00 | 1,92E-02 | | | #NUM! | FCGR3A/FCGR3B,MIF,NGFR,SFRP4,SOCS3,TNFRSF11B |
| Role of Pattern Recognition Receptors in Recognition of Bacteria and Viruses | 2,01E00 | 2,6E-02 | | | #NUM! | C1QA,C1QB,C1QC,TGFB2 |
| Allograft Rejection Signaling | 1,94E00 | 3,49E-02 | | | #NUM! | HLA-DPA1,HLA-DRA,HLA-DRB1 |
| Crosstalk between Dendritic Cells and Natural Killer Cells | 1,9E00 | 3,37E-02 | | | #NUM! | ACTC1,HLA-DRA,HLA-DRB1 |
| OX40 Signaling Pathway | 1,89E00 | 3,33E-02 | | | #NUM! | HLA-DPA1,HLA-DRA,HLA-DRB1 |
| Tight Junction Signaling | 1,88E00 | 2,38E-02 | | | #NUM! | ACTC1,NGFR,TGFB2,TNFRSF11B |
| Th1 and Th2 Activation Pathway | 1,85E00 | 2,34E-02 | | | #NUM! | HLA-DPA1,HLA-DRA,HLA-DRB1,SOCS3 |
| MIF-mediated Glucocorticoid Regulation | 1,84E00 | 5,88E-02 | | | #NUM! | MIF,PTGS2 |
| B Cell Development | 1,79E00 | 5,56E-02 | | | #NUM! | HLA-DRA,HLA-DRB1 |
| Acute Phase Response Signaling | 1,78E00 | 2,22E-02 | | | 0,000 | A2M,NGFR,SOCS3,TNFRSF11B |
| Hepatic Fibrosis / Hepatic Stellate Cell Activation | 1,73E00 | 2,15E-02 | | | #NUM! | A2M,NGFR,TGFB2,TNFRSF11B |
| Antiproliferative Role of TOB in T Cell Signaling | 1,72E00 | 5,13E-02 | | | #NUM! | DPP7,TGFB2 |
| Production of Nitric Oxide and Reactive Oxygen Species in Macrophages | 1,71E00 | 2,12E-02 | | | -1,000 | CYBA,LYZ,NGFR,TNFRSF11B |
| CDK5 Signaling | 1,68E00 | 2,78E-02 | | | #NUM! | FOSB,NGF,NGFR |
| Sirtuin Signaling Pathway | 1,67E00 | 1,72E-02 | | | #NUM! | ABCA1,ATP5F1D,NDUFB7,NDUFS7,TSPO |
| Oxidative Phosphorylation | 1,67E00 | 2,75E-02 | | | #NUM! | ATP5F1D,NDUFB7,NDUFS7 |
| MIF Regulation of Innate Immunity | 1,66E00 | 4,76E-02 | | | #NUM! | MIF,PTGS2 |
| Neuroinflammation Signaling Pathway | 1,63E00 | 1,67E-02 | | | -1,342 | HLA-DRA,HLA-DRB1,NGF,PTGS2,TGFB2 |
| Creatine-phosphate Biosynthesis | 1,57E00 | 0,2 | | | #NUM! | CKB |
| Serine Biosynthesis | 1,57E00 | 0,2 | | | #NUM! | PSAT1 |
| Eumelanin Biosynthesis | 1,57E00 | 0,2 | | | #NUM! | MIF |
| Graft-versus-Host Disease Signaling | 1,55E00 | 4,17E-02 | | | #NUM! | HLA-DRA,HLA-DRB1 |
| Autoimmune Thyroid Disease Signaling | 1,54E00 | 4,08E-02 | | | #NUM! | HLA-DRA,HLA-DRB1 |
| Myc Mediated Apoptosis Signaling | 1,52E00 | 0,04 | | | #NUM! | NGFR,TNFRSF11B |
| Atherosclerosis Signaling | 1,5E00 | 2,36E-02 | | | #NUM! | F3,LPL,LYZ |
| LPS/IL-1 Mediated Inhibition of RXR Function | 1,47E00 | 1,78E-02 | | | #NUM! | ABCA1,FMO2,NGFR,TNFRSF11B |
| STAT3 Pathway | 1,43E00 | 2,22E-02 | | | #NUM! | NGFR,SOCS3,TGFB2 |
| Superpathway of Serine and Glycine Biosynthesis I | 1,43E00 | 1,43E-01 | | | #NUM! | PSAT1 |
| Glucocorticoid Receptor Signaling | 1,4E00 | 1,3E-02 | | | #NUM! | A2M,ATP5F1D,CD163,KRT19,PTGS2,TGFB2 |
| Type II Diabetes Mellitus Signaling | 1,38E00 | 2,11E-02 | | | #NUM! | NGFR,SOCS3,TNFRSF11B |
| Salvage Pathways of Pyrimidine Deoxyribonucleotides | 1,37E00 | 1,25E-01 | | | #NUM! | TK1 |
| Induction of Apoptosis by HIV1 | 1,36E00 | 3,28E-02 | | | #NUM! | NGFR,TNFRSF11B |
| Calcium-induced T Lymphocyte Apoptosis | 1,3E00 | 3,03E-02 | | | #NUM! | HLA-DRA,HLA-DRB1 |
| Mitotic Roles of Polo-Like Kinase | 1,3E00 | 3,03E-02 | | | #NUM! | CDC20,PKMYT1 |
| Eicosanoid Signaling | 1,3E00 | 3,03E-02 | | | #NUM! | PTGFR,PTGS2 |
| Prostanoid Biosynthesis | 1,28E00 | 0,1 | | | #NUM! | PTGS2 |
| Necroptosis Signaling Pathway | 1,27E00 | 1,91E-02 | | | #NUM! | NGFR,TNFRSF11B,TSPO |
| Growth Hormone Signaling | 1,25E00 | 2,82E-02 | | | #NUM! | A2M,SOCS3 |
| Caveolar-mediated Endocytosis Signaling | 1,23E00 | 2,74E-02 | | | #NUM! | ACTC1,ITGA8 |
| HMGB1 Signaling | 1,22E00 | 1,82E-02 | | | #NUM! | NGFR,TGFB2,TNFRSF11B |
| Neurotrophin/TRK Signaling | 1,19E00 | 2,63E-02 | | | #NUM! | NGF,NGFR |
| Macropinocytosis Signaling | 1,19E00 | 2,63E-02 | | | #NUM! | MRC1,NGF |
| Mitochondrial Dysfunction | 1,18E00 | 1,75E-02 | | | #NUM! | ATP5F1D,NDUFB7,NDUFS7 |
| Germ Cell-Sertoli Cell Junction Signaling | 1,18E00 | 1,75E-02 | | | #NUM! | A2M,ACTC1,TGFB2 |
| Oleate Biosynthesis II (Animals) | 1,17E00 | 7,69E-02 | | | #NUM! | SCD |
| BEX2 Signaling Pathway | 1,16E00 | 2,53E-02 | | | #NUM! | NGF,NGFR |
| T Cell Exhaustion Signaling Pathway | 1,16E00 | 1,71E-02 | | | #NUM! | HLA-DPA1,HLA-DRA,HLA-DRB1 |
| Cdc42 Signaling | 1,15E00 | 1,7E-02 | | | #NUM! | HLA-DPA1,HLA-DRA,HLA-DRB1 |
| NF-κB Signaling | 1,13E00 | 1,68E-02 | | | #NUM! | NGF,NGFR,TNFRSF11B |
| Role of NFAT in Regulation of the Immune Response | 1,12E00 | 1,66E-02 | | | #NUM! | FCGR3A/FCGR3B,HLA-DRA,HLA-DRB1 |
| IL-4 Signaling | 1,11E00 | 2,35E-02 | | | #NUM! | HLA-DRA,HLA-DRB1 |
| Hepatic Cholestasis | 1,1E00 | 1,61E-02 | | | #NUM! | NGFR,TGFB2,TNFRSF11B |
| Regulation Of The Epithelial Mesenchymal Transition By Growth Factors Pathway | 1,08E00 | 1,6E-02 | | | #NUM! | NGFR,TGFB2,TNFRSF11B |
| Ceramide Signaling | 1,08E00 | 2,27E-02 | | | #NUM! | NGFR,TNFRSF11B |
| PPARα/RXRα Activation | 1,07E00 | 1,57E-02 | | | #NUM! | ABCA1,LPL,TGFB2 |
| Altered T Cell and B Cell Signaling in Rheumatoid Arthritis | 1,07E00 | 2,22E-02 | | | #NUM! | HLA-DRA,HLA-DRB1 |
| Leukocyte Extravasation Signaling | 1,06E00 | 1,55E-02 | | | #NUM! | ACTC1,CYBA,EDIL3 |
| Fcγ Receptor-mediated Phagocytosis in Macrophages and Monocytes | 1,04E00 | 2,13E-02 | | | #NUM! | ACTC1,FCGR3A/FCGR3B |
| Communication between Innate and Adaptive Immune Cells | 1,02E00 | 2,08E-02 | | | #NUM! | HLA-DRA,HLA-DRB1 |
| VEGF Signaling | 9,96E-01 | 2,02E-02 | | | #NUM! | ACTC1,EIF1AY |
| Fatty Acid α-oxidation | 9,87E-01 | 0,05 | | | #NUM! | PTGS2 |
| Kinetochore Metaphase Signaling Pathway | 9,83E-01 | 1,98E-02 | | | #NUM! | CDC20,H2AX |
| Pyrimidine Deoxyribonucleotides De Novo Biosynthesis I | 9,47E-01 | 4,55E-02 | | | #NUM! | NME3 |
| Role of Osteoblasts, Osteoclasts and Chondrocytes in Rheumatoid Arthritis | 9,39E-01 | 1,38E-02 | | | #NUM! | NGFR,SFRP4,TNFRSF11B |
| Paxillin Signaling | 9,36E-01 | 1,85E-02 | | | #NUM! | ACTC1,ITGA8 |
| Apelin Cardiac Fibroblast Signaling Pathway | 9,32E-01 | 4,35E-02 | | | #NUM! | TGFB2 |
| Pancreatic Adenocarcinoma Signaling | 9,28E-01 | 1,83E-02 | | | #NUM! | PTGS2,TGFB2 |
| iCOS-iCOSL Signaling in T Helper Cells | 9,17E-01 | 1,8E-02 | | | #NUM! | HLA-DRA,HLA-DRB1 |
| IL-22 Signaling | 9,14E-01 | 4,17E-02 | | | #NUM! | SOCS3 |
| EIF2 Signaling | 9,14E-01 | 1,34E-02 | | | #NUM! | ACTC1,EIF1AY,RPS4Y1 |
| Role of JAK family kinases in IL-6-type Cytokine Signaling | 8,96E-01 | 0,04 | | | #NUM! | SOCS3 |
| NGF Signaling | 8,96E-01 | 1,75E-02 | | | #NUM! | NGF,NGFR |
| Axonal Guidance Signaling | 8,86E-01 | 1,01E-02 | | | #NUM! | ADAMDEC1,ADAMTS9,NGF,NGFR,PAPPA |
| Cardiac Hypertrophy Signaling (Enhanced) | 8,79E-01 | 1,01E-02 | | | 0,447 | HAND2,NGFR,PTGS2,TGFB2,TNFRSF11B |
| CD28 Signaling in T Helper Cells | 8,54E-01 | 1,65E-02 | | | #NUM! | HLA-DRA,HLA-DRB1 |
| Inhibition of ARE-Mediated mRNA Degradation Pathway | 8,51E-01 | 1,64E-02 | | | #NUM! | NGFR,TNFRSF11B |
| Airway Inflammation in Asthma | 7,96E-01 | 3,12E-02 | | | #NUM! | TGFB2 |
| IL-12 Signaling and Production in Macrophages | 7,9E-01 | 1,5E-02 | | | #NUM! | LYZ,TGFB2 |
| Phagosome Formation | 7,9E-01 | 1,5E-02 | | | #NUM! | FCGR3A/FCGR3B,MRC1 |
| IL-9 Signaling | 7,85E-01 | 3,03E-02 | | | #NUM! | SOCS3 |
| Human Embryonic Stem Cell Pluripotency | 7,8E-01 | 1,48E-02 | | | #NUM! | NGF,TGFB2 |
| Role of JAK2 in Hormone-like Cytokine Signaling | 7,72E-01 | 2,94E-02 | | | #NUM! | SOCS3 |
| DNA Methylation and Transcriptional Repression Signaling | 7,62E-01 | 2,86E-02 | | | #NUM! | MBD3 |
| IL-17A Signaling in Fibroblasts | 7,62E-01 | 2,86E-02 | | | #NUM! | NFKBIZ |
| Protein Ubiquitination Pathway | 7,35E-01 | 1,1E-02 | | | #NUM! | CDC20,USP53,USP9Y |
| Inhibition of Matrix Metalloproteases | 7,19E-01 | 2,56E-02 | | | #NUM! | A2M |
| Coronavirus Pathogenesis Pathway | 7,1E-01 | 1,33E-02 | | | #NUM! | PTGS2,RPS4Y1 |
| Phagosome Maturation | 7,06E-01 | 1,32E-02 | | | #NUM! | HLA-DRA,HLA-DRB1 |
| Epithelial Adherens Junction Signaling | 7,01E-01 | 1,32E-02 | | | #NUM! | ACTC1,TGFB2 |
| Mechanisms of Viral Exit from Host Cells | 6,99E-01 | 2,44E-02 | | | #NUM! | ACTC1 |
| Intrinsic Prothrombin Activation Pathway | 6,9E-01 | 2,38E-02 | | | #NUM! | F13A1 |
| Pyrimidine Ribonucleotides Interconversion | 6,9E-01 | 2,38E-02 | | | #NUM! | NME3 |
| PKCθ Signaling in T Lymphocytes | 6,88E-01 | 1,29E-02 | | | #NUM! | HLA-DRA,HLA-DRB1 |
| Oncostatin M Signaling | 6,82E-01 | 2,33E-02 | | | #NUM! | MT2A |
| Retinol Biosynthesis | 6,82E-01 | 2,33E-02 | | | #NUM! | LPL |
| Pyrimidine Ribonucleotides De Novo Biosynthesis | 6,72E-01 | 2,27E-02 | | | #NUM! | NME3 |
| IL-23 Signaling Pathway | 6,72E-01 | 2,27E-02 | | | #NUM! | SOCS3 |
| PFKFB4 Signaling Pathway | 6,56E-01 | 2,17E-02 | | | #NUM! | TGFB2 |
| Ephrin A Signaling | 6,48E-01 | 2,13E-02 | | | #NUM! | NGFR |
| Regulation of eIF4 and p70S6K Signaling | 6,44E-01 | 1,2E-02 | | | #NUM! | EIF1AY,RPS4Y1 |
| Triacylglycerol Degradation | 6,31E-01 | 2,04E-02 | | | #NUM! | LPL |
| Cell Cycle: G2/M DNA Damage Checkpoint Regulation | 6,31E-01 | 2,04E-02 | | | #NUM! | PKMYT1 |
| Granulocyte Adhesion and Diapedesis | 6,22E-01 | 1,16E-02 | | | #NUM! | NGFR,TNFRSF11B |
| Wnt/β-catenin Signaling | 6,18E-01 | 1,16E-02 | | | #NUM! | SFRP4,TGFB2 |
| Tumor Microenvironment Pathway | 6,07E-01 | 1,14E-02 | | | #NUM! | PTGS2,TGFB2 |
| Role of IL-17A in Arthritis | 5,95E-01 | 1,85E-02 | | | #NUM! | PTGS2 |
| Cell Cycle Control of Chromosomal Replication | 5,82E-01 | 1,79E-02 | | | #NUM! | CDT1 |
| Glutamate Receptor Signaling | 5,75E-01 | 1,75E-02 | | | #NUM! | GRIK2 |
| MSP-RON Signaling Pathway | 5,69E-01 | 1,72E-02 | | | #NUM! | ACTC1 |
| Cancer Drug Resistance By Drug Efflux | 5,69E-01 | 1,72E-02 | | | #NUM! | PTGS2 |
| Systemic Lupus Erythematosus In T Cell Signaling Pathway | 5,67E-01 | 8,98E-03 | | | #NUM! | HLA-DPA1,HLA-DRA,HLA-DRB1 |
| Xenobiotic Metabolism CAR Signaling Pathway | 5,64E-01 | 1,06E-02 | | | #NUM! | FMO2,SCAND1 |
| ILK Signaling | 5,61E-01 | 1,05E-02 | | | #NUM! | ACTC1,PTGS2 |
| Maturity Onset Diabetes of Young (MODY) Signaling | 5,56E-01 | 1,67E-02 | | | #NUM! | APOL6 |
| Clathrin-mediated Endocytosis Signaling | 5,51E-01 | 1,04E-02 | | | #NUM! | ACTC1,LYZ |
| RAR Activation | 5,48E-01 | 1,03E-02 | | | #NUM! | SCAND1,TGFB2 |
| Sertoli Cell-Sertoli Cell Junction Signaling | 5,48E-01 | 1,03E-02 | | | #NUM! | A2M,ACTC1 |
| Gap Junction Signaling | 5,36E-01 | 1,01E-02 | | | #NUM! | ACTC1,GRIK2 |
| PXR/RXR Activation | 5,26E-01 | 1,54E-02 | | | #NUM! | SCD |
| CD40 Signaling | 5,26E-01 | 1,54E-02 | | | #NUM! | PTGS2 |
| Phospholipases | 5,26E-01 | 1,54E-02 | | | #NUM! | LPL |
| Nicotine Degradation II | 5,26E-01 | 1,54E-02 | | | #NUM! | FMO2 |
| HIF1α Signaling | 5,16E-01 | 9,76E-03 | | | #NUM! | SLC2A5,TGFB2 |
| Cell Cycle: G1/S Checkpoint Regulation | 5,16E-01 | 1,49E-02 | | | #NUM! | TGFB2 |
| Remodeling of Epithelial Adherens Junctions | 5,1E-01 | 1,47E-02 | | | #NUM! | ACTC1 |
| Role of JAK1 and JAK3 in γc Cytokine Signaling | 5,04E-01 | 1,45E-02 | | | #NUM! | SOCS3 |
| SPINK1 General Cancer Pathway | 5,04E-01 | 1,45E-02 | | | #NUM! | MT2A |
| IL-10 Signaling | 0,5 | 1,43E-02 | | | #NUM! | SOCS3 |
| Agrin Interactions at Neuromuscular Junction | 0,5 | 1,43E-02 | | | #NUM! | ACTC1 |
| Small Cell Lung Cancer Signaling | 4,95E-01 | 1,41E-02 | | | #NUM! | PTGS2 |
| Integrin Signaling | 4,93E-01 | 9,39E-03 | | | #NUM! | ACTC1,ITGA8 |
| ERK5 Signaling | 4,89E-01 | 1,39E-02 | | | #NUM! | NGF |
| Leptin Signaling in Obesity | 4,8E-01 | 1,35E-02 | | | #NUM! | SOCS3 |
| Hepatic Fibrosis Signaling Pathway | 4,78E-01 | 7,98E-03 | | | #NUM! | NGFR,TGFB2,TNFRSF11B |
| VDR/RXR Activation | 4,61E-01 | 1,28E-02 | | | #NUM! | TGFB2 |
| Thyroid Cancer Signaling | 4,57E-01 | 1,27E-02 | | | #NUM! | NGF |
| Systemic Lupus Erythematosus Signaling | 4,53E-01 | 8,73E-03 | | | #NUM! | C7,FCGR3A/FCGR3B |
| IL-17 Signaling | 4,52E-01 | 1,25E-02 | | | #NUM! | PTGS2 |
| Role of MAPK Signaling in the Pathogenesis of Influenza | 4,52E-01 | 1,25E-02 | | | #NUM! | PTGS2 |
| JAK/Stat Signaling | 4,52E-01 | 1,25E-02 | | | #NUM! | SOCS3 |
| Prolactin Signaling | 4,49E-01 | 1,23E-02 | | | #NUM! | SOCS3 |
| Cyclins and Cell Cycle Regulation | 4,49E-01 | 1,23E-02 | | | #NUM! | TGFB2 |
| PEDF Signaling | 4,44E-01 | 1,22E-02 | | | #NUM! | NGF |
| Protein Kinase A Signaling | 4,35E-01 | 7,52E-03 | | | #NUM! | NGFR,PTGS2,TGFB2 |
| Regulation Of The Epithelial Mesenchymal Transition In Development Pathway | 4,35E-01 | 1,19E-02 | | | #NUM! | S100A4 |
| Huntington's Disease Signaling | 4,34E-01 | 8,44E-03 | | | #NUM! | ATP5F1D,NGF |
| Cardiac Hypertrophy Signaling | 4,27E-01 | 8,33E-03 | | | #NUM! | HAND2,TGFB2 |
| Regulation of IL-2 Expression in Activated and Anergic T Lymphocytes | 4,12E-01 | 1,11E-02 | | | #NUM! | TGFB2 |
| Th17 Activation Pathway | 4,09E-01 | 1,1E-02 | | | #NUM! | SOCS3 |
| Death Receptor Signaling | 4,05E-01 | 1,09E-02 | | | #NUM! | ACTC1 |
| Colorectal Cancer Metastasis Signaling | 3,99E-01 | 7,91E-03 | | | #NUM! | PTGS2,TGFB2 |
| CREB Signaling in Neurons | 3,93E-01 | 6,7E-03 | | | 0,000 | GRIK2,NGFR,PTGFR,TGFB2 |
| TGF-β Signaling | 3,9E-01 | 1,04E-02 | | | #NUM! | TGFB2 |
| Amyotrophic Lateral Sclerosis Signaling | 3,87E-01 | 1,03E-02 | | | #NUM! | GRIK2 |
| ATM Signaling | 3,87E-01 | 1,03E-02 | | | #NUM! | H2AX |
| Salvage Pathways of Pyrimidine Ribonucleotides | 3,84E-01 | 1,02E-02 | | | #NUM! | NME3 |
| Virus Entry via Endocytic Pathways | 3,71E-01 | 9,8E-03 | | | #NUM! | ACTC1 |
| Regulation of Actin-based Motility by Rho | 3,68E-01 | 9,71E-03 | | | #NUM! | ACTC1 |
| Chronic Myeloid Leukemia Signaling | 3,68E-01 | 9,71E-03 | | | #NUM! | TGFB2 |
| FAK Signaling | 3,65E-01 | 9,62E-03 | | | #NUM! | ACTC1 |
| IGF-1 Signaling | 3,65E-01 | 9,62E-03 | | | #NUM! | SOCS3 |
| Antioxidant Action of Vitamin C | 3,5E-01 | 9,17E-03 | | | #NUM! | SLC2A5 |
| Xenobiotic Metabolism Signaling | 3,36E-01 | 6,97E-03 | | | #NUM! | FMO2,SCAND1 |
| Neuroprotective Role of THOP1 in Alzheimer's Disease | 3,3E-01 | 8,62E-03 | | | #NUM! | CFD |
| Role of Tissue Factor in Cancer | 3,3E-01 | 8,62E-03 | | | #NUM! | F3 |
| Airway Pathology in Chronic Obstructive Pulmonary Disease | 3,24E-01 | 8,47E-03 | | | #NUM! | TGFB2 |
| p38 MAPK Signaling | 3,24E-01 | 8,47E-03 | | | #NUM! | TGFB2 |
| Cholecystokinin/Gastrin-mediated Signaling | 3,21E-01 | 8,4E-03 | | | #NUM! | PTGS2 |
| HGF Signaling | 3,2E-01 | 8,33E-03 | | | #NUM! | PTGS2 |
| RhoA Signaling | 3,12E-01 | 8,13E-03 | | | #NUM! | ACTC1 |
| FXR/RXR Activation | 3,05E-01 | 7,94E-03 | | | #NUM! | LPL |
| Endocannabinoid Neuronal Synapse Pathway | 2,99E-01 | 7,81E-03 | | | #NUM! | PTGS2 |
| Cellular Effects of Sildenafil (Viagra) | 2,92E-01 | 7,63E-03 | | | #NUM! | ACTC1 |
| Adipogenesis pathway | 2,86E-01 | 7,46E-03 | | | #NUM! | LPL |
| PTEN Signaling | 2,81E-01 | 7,35E-03 | | | #NUM! | NGFR |
| Iron homeostasis signaling pathway | 2,79E-01 | 7,3E-03 | | | #NUM! | CD163 |
| Ovarian Cancer Signaling | 2,75E-01 | 7,19E-03 | | | #NUM! | PTGS2 |
| Hereditary Breast Cancer Signaling | 2,73E-01 | 7,14E-03 | | | #NUM! | H2AX |
| Insulin Receptor Signaling | 2,73E-01 | 7,14E-03 | | | #NUM! | SOCS3 |
| D-myo-inositol (1,4,5,6)-Tetrakisphosphate Biosynthesis | 2,69E-01 | 7,04E-03 | | | #NUM! | SOCS3 |
| D-myo-inositol (3,4,5,6)-tetrakisphosphate Biosynthesis | 2,69E-01 | 7,04E-03 | | | #NUM! | SOCS3 |
| Aryl Hydrocarbon Receptor Signaling | 2,67E-01 | 6,99E-03 | | | #NUM! | TGFB2 |
| Corticotropin Releasing Hormone Signaling | 2,63E-01 | 6,9E-03 | | | #NUM! | PTGS2 |
| Factors Promoting Cardiogenesis in Vertebrates | 2,53E-01 | 6,67E-03 | | | #NUM! | TGFB2 |
| 3-phosphoinositide Degradation | 2,43E-01 | 6,41E-03 | | | #NUM! | SOCS3 |
| D-myo-inositol-5-phosphate Metabolism | 2,4E-01 | 6,37E-03 | | | #NUM! | SOCS3 |
| eNOS Signaling | 2,37E-01 | 6,29E-03 | | | #NUM! | AQP1 |
| 3-phosphoinositide Biosynthesis | 2,25E-01 | 6,02E-03 | | | #NUM! | SOCS3 |
| Erythropoietin Signaling | 2,15E-01 | 5,78E-03 | | | #NUM! | TGFB2 |
| Tec Kinase Signaling | 2,15E-01 | 5,78E-03 | | | #NUM! | ACTC1 |
| Natural Killer Cell Signaling | 0 | 5,08E-03 | | | #NUM! | FCGR3A/FCGR3B |
| Actin Cytoskeleton Signaling | 0 | 4,41E-03 | | | #NUM! | ACTC1 |
| NRF2-mediated Oxidative Stress Response | 0 | 5,29E-03 | | | #NUM! | ACTC1 |
| IL-8 Signaling | 0 | 0,005 | | | #NUM! | PTGS2 |
| Endothelin-1 Signaling | 0 | 5,32E-03 | | | #NUM! | PTGS2 |
| Molecular Mechanisms of Cancer | 0 | 2,5E-03 | | | #NUM! | TGFB2 |
| mTOR Signaling | 0 | 4,76E-03 | | | #NUM! | RPS4Y1 |
| Role of NFAT in Cardiac Hypertrophy | 0 | 4,67E-03 | | | #NUM! | TGFB2 |
| Breast Cancer Regulation by Stathmin1 | 0 | 3,38E-03 | | | #NUM! | PTGFR,TGFB2 |
| Signaling by Rho Family GTPases | 0 | 3,95E-03 | | | #NUM! | ACTC1 |
| RhoGDI Signaling | 0 | 5,29E-03 | | | #NUM! | ACTC1 |
| Superpathway of Inositol Phosphate Compounds | 0 | 5,03E-03 | | | #NUM! | SOCS3 |
| Agranulocyte Adhesion and Diapedesis | 0 | 5,21E-03 | | | #NUM! | ACTC1 |
| Regulation of the Epithelial-Mesenchymal Transition Pathway | 0 | 5,21E-03 | | | #NUM! | TGFB2 |
| Estrogen Receptor Signaling | 0 | 3,05E-03 | | | #NUM! | ATP5F1D |
| PI3K/AKT Signaling | 0 | 5,41E-03 | | | #NUM! | PTGS2 |
| Osteoarthritis Pathway | 0 | 4,55E-03 | | | #NUM! | PTGS2 |
| Opioid Signaling Pathway | 0 | 4,05E-03 | | | #NUM! | FOSB |
| Synaptogenesis Signaling Pathway | 0 | 3,21E-03 | | | #NUM! | NLGN4Y |
| Systemic Lupus Erythematosus In B Cell Signaling Pathway | 0 | 3,64E-03 | | | #NUM! | TGFB2 |
| Senescence Pathway | 0 | 3,64E-03 | | | #NUM! | TGFB2 |
| Xenobiotic Metabolism PXR Signaling Pathway | 0 | 5,21E-03 | | | #NUM! | SCAND1 |
| Insulin Secretion Signaling Pathway | 0 | 4,1E-03 | | | #NUM! | SLC2A5 |
| Calcium Signaling | 0 | 4,85E-03 | | | #NUM! | ACTC1 |
